# Partitioning of respired CO_2_ in newly sprouted Moso bamboo culms

**DOI:** 10.3389/fpls.2023.1154232

**Published:** 2023-04-20

**Authors:** Chongyu Ye, Qiangfa Zeng, Keda Hu, Dongming Fang, Dirk Hölscher, Huaqiang Du, Yongjun Shi, Yufeng Zhou, Frank Berninger, Tingting Mei, Guomo Zhou

**Affiliations:** ^1^ State Key Laboratory of Subtropical Silviculture, Zhejiang A&F University, Lin’an, Zhejiang, China; ^2^ Key Laboratory of Carbon Cycling in Forest Ecosystems and Carbon Sequestration of Zhejiang Province, Zhejiang A&F University, Lin’an, Zhejiang, China; ^3^ College of Environmental and Resource Sciences, Zhejiang A&F University, Lin’an, Zhejiang, China; ^4^ Jiyang College, Zhejiang A&F University, Zhuji, Zhejiang, China; ^5^ Tropical Silviculture and Forest Ecology, University of Göttingen, Göttingen, Germany; ^6^ Department of Environmental and Biological Sciences, University of Eastern Finland, Joensuu, Finland

**Keywords:** explosive growth, stem respiration, sap flow, leaf phenology, stem photosynthesis

## Abstract

Stem respiration (*R*_s_) plays a vital role in ecosystem carbon cycling. However, the measured efflux on the stem surface (*E*_s_) is not always *in situ R*_s_ but only part of it. A previously proposed mass balance framework (MBF) attempted to explore the multiple partitioning pathways of *R*_s_, including sap-flow-transported and internal storage of *R*_s,_ in addition to *E*_s_. This study proposed stem photosynthesis as an additional partitioning pathway to the MBF. Correspondingly, a double-chamber apparatus was designed and applied on newly sprouted Moso bamboo (*Phyllostachys edulis*) in leafless and leaved stages. *R*_s_ of newly sprouted bamboo were twice as high in the leafless stage (7.41 ± 2.66 μmol m^−2^ s^−1^) than in the leaved stage (3.47 ± 2.43 μmol m^−2^ s^−1^). *E*_s_ accounted for ~80% of *R*_s,_ while sap flow may take away ~2% of *R*_s_ in both leafless and leaved stages. Culm photosynthesis accounted for ~9% and 13% of *R*_s_, respectively. Carbon sequestration from culm photosynthesis accounted for approximately 2% of the aboveground bamboo biomass in the leafless stage. High culm photosynthesis but low sap flow during the leafless stage and vice versa during the leaved stage make bamboo an outstanding choice for exploring the MBF.

## 1 Introduction

Stem respiration (*R*_s_) is a complex physiological process involving enzyme-catalyzed reactions, which is thought to be mainly influenced by temperature (Amthor, 2000; Atkin et al., 2005). We synthesized and analyzed measured stem-surface CO_2_ efflux (*E*_s_) of 191 woody plant species from 66 published papers, and the results confirmed the significant positive effects of temperature on *E*_s_ (*P* < 0.01; **Figure S1**). Still, we found the temperature can only explain 20–42% variation of *R*_s_, which corroborated the varying explaining power of temperature (17–75%) to the variance of stem respiration in other studies (Zhu et al., 2012). Furtherly, several studies observed the decoupling between respiration and temperature, including the time lag between respiration and temperature (Saveyn et al., 2008), the “midday depression” of stem respiration (Saveyn et al., 2008). One of the hypotheses explaining this decoupling is that the measured *E*_s_ was not *in situ* but apparent *R*_s_ (Ceschia et al., 2002; McGuire and Teskey, 2002; McGuire and Teskey, 2004), i.e., *E*_s_ is only one part of *R*_s_. The gap between *R*_s_ and *E*_s_ may suggest some missing efflux (*E*_miss_) being ignored by the conventional approach. Therefore, a mass balance framework (MBF) on *R*_s_ was proposed by McGuire and Teskey (2004) based on previous ideas and observations (McGuire and Teskey, 2002).

According to the MBF, CO_2_ produced by *R*_s_ at a given position of a stem is allocated into three pathways: 1) CO_2_ efflux released from stem surface, i.e., *E*_s_, 2) CO_2_ efflux transported in sap flow (*E*_T_), and 3) CO_2_ efflux stored internally (*E*_I_). Therefore, *E*_miss_ includes both *E*_T_ and *E*_I_. Compared with *E*_s_ measured outside a stem, the other two parts are more challenging to detect and quantify. Based on the MBF, CO_2_ released by respiring cells in woody tissues could be dissolved in xylem sap and transported upward by sap rather than diffusing into the atmosphere directly (McGuire and Teskey, 2004; Teskey et al., 2007; Aubrey and Teskey, 2009). The isotopic tracing method (^14^C or ^13^C) proved the existence of *E*_T_ on some trees (Bloemen et al., 2013; Salomón et al., 2019; Salomón et al., 2020). Moreover, up to 17% of the upward transported *E*_T_ could be refixed by leaf photosynthesis (Bloemen et al., 2013). In contrast, *E*_I_ was limited by CO_2_ saturation in sap according to equilibrium reactions, which were pH-dependent and more challenging to measure *in situ* (Teskey et al., 2007).

**Figure 1:**
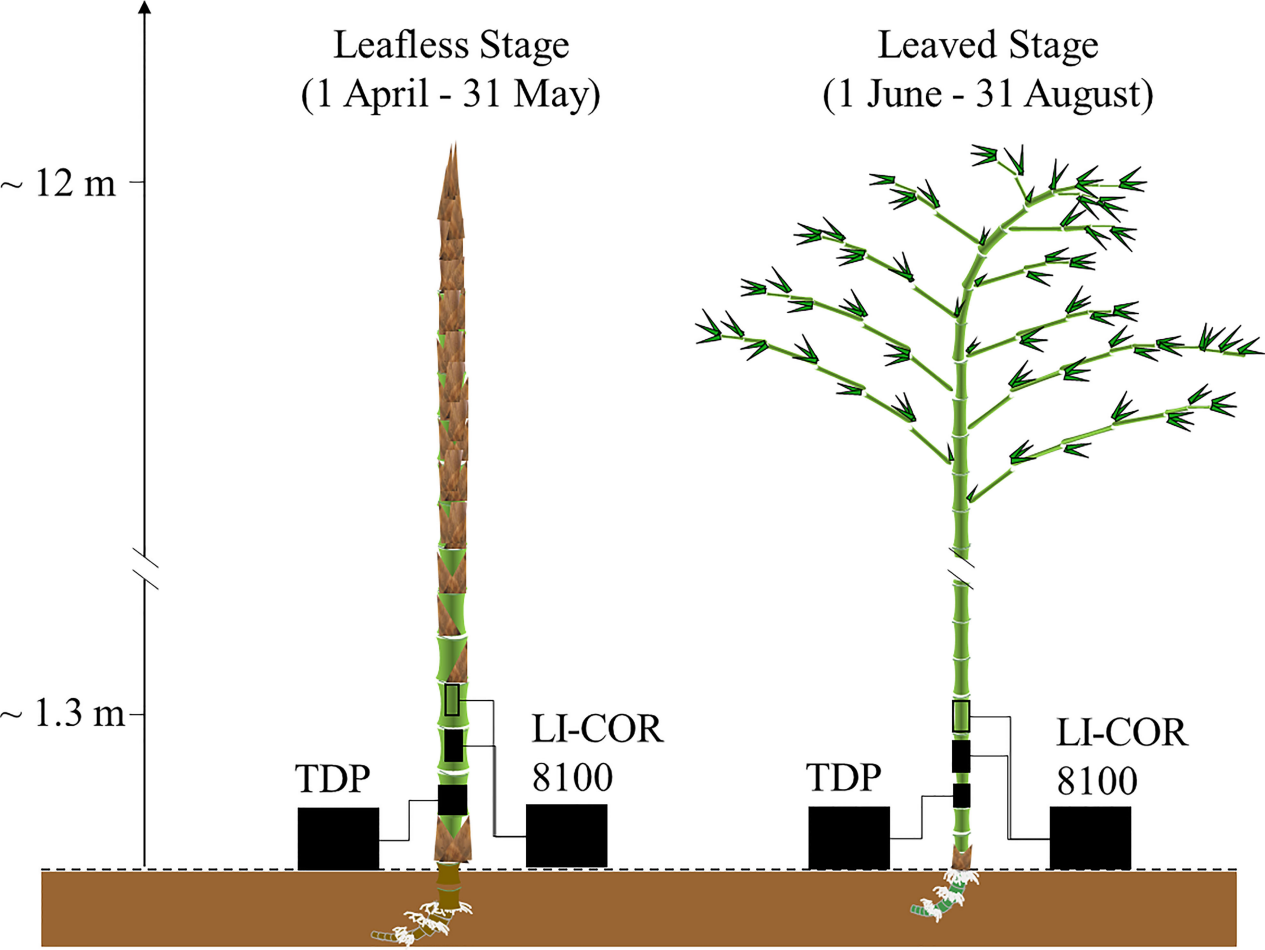
Bamboo phenology and experimental design. This study monitored sap flow with a self-built thermal dissipation probe based on the classical Granier type (TDP). In addition, CO_2_ efflux in the field was measured with LI-COR 8100 (Li-cor Inc., USA) using transparent and light-proof chambers.

Besides, in some species with abundant stem chloroplasts, a fourth partitioning pathway was proposed, i.e., the CO_2_ reused by the culm/stem photosynthesis (*E*_p_) (Pfanz et al., 2002; Wittmann et al., 2006; Berveiller et al., 2007; Teskey et al., 2007; Cernusak and Cheesman, 2015; Wittmann and Pfanz, 2018). *E*_p_ was confirmed with measured chlorophyll fluorescence on six coniferous and two broad-leaved tree species (Berveiller et al., 2007) and with an isotope tracing method on several C3 and CAM species (Kocurek et al., 2015). Furthermore, *E*_p_ was estimated continuously by comparing the theoretical *R*_s_ derived from stem temperature and *E*_s_ on boreal Scots pines (*Pinus sylvestris* L.; Tarvainen et al., 2018). However, such a method of calculating *E*_p_ might still overestimate *E*_p_ when ignoring *E*_T_ and *E*_I_. To resolve this problem in this study, we proposed an experimental apparatus including a pair of simultaneously monitoring chambers (one transparent and another light-proof; **Figure 1**). By applying one pair of chambers on the same stem and comparing the difference of *R*_s_ and *E*_s_ between the chambers, we could derive *E*_p_ (see Section 2.3). In this way, we could avoid measuring *E*_T_ and *E*_I_ *in situ*, which is difficult to accurately measure due to the current limited techniques.

To test the designed method, we chose Moso bamboo (*Phyllostachys edulis* (Carriere) J. Houzeau) as the experimental object. Unlike tree stems, bamboo culm has a smooth circular surface and a hollow cavity of each internode. Such characteristics make monitoring and measuring efflux pathways much easier and more accessible. Firstly, the traditional chambers measuring *E*_s_ (Zhu et al., 2012; Darenova et al., 2018; Katayama et al., 2019; Helm et al., 2021) could be easily attached to the smooth circular bamboo culm surface with better sealing. Secondly, as the hollow cavity occupies the most volume of the internode, the thin culm wall (~1 cm thickness in an internode with 10 cm diameter at breast height) may have limited space for *E*_I_, which may reduce the disturbance for estimating the other allocated CO_2_ efflux (e.g., *E*_T_ and *E*_p_). At last, the observed large amount of chlorophyll in bamboo culms (Wang et al., 2013) implied the existence of *E*_p_, thus making a possible examination of the four pathways of the MBF, which suggested Moso bamboo could be a fantastic model plant for exploring MBF.

As the most distributed bamboo species in China (National Forestry and Grassland Administration, 2019; FAO, 2020), Moso bamboo grows very fast, taking only 6–8 weeks for a newly sprouted culm to finish its ~12 m height growth; such a process was named “explosive growth” (Wang et al., 2019; Mei et al., 2020; Li et al., 2022). Especially the newly sprouted culms finish their height growth without leaves, which means they may primarily rely on an external supply of water (Fang et al., 2019; Gu et al., 2019; Wu et al., 2019; Mei et al., 2020) and carbon (Wei et al., 2019; Li et al., 2022) from other established elder culms in the leafless stage. The previous studies found that the water use patterns in the leafless and leaved stages were opposite (Fang et al., 2019; Tong et al., 2021), characterizing peak values of the sap flux density at midnight and midday, respectively. As the newly expanded leaves will lead to increasing transpiration and sap flow in the culms, we hypothesized our first assumption that sap flow might take away more CO_2_ efflux and influence more negatively to *E*_s_ in the leaved stage than in the leafless stage. Additionally, the increasing *E*_T_ may decrease the effect of culm photosynthesis. Thus, our second assumption was that *E*_p_ played a more significant role in the leafless stage than in the leaved stage. In summary, MBF theory was tested with a two-chambers experimental apparatus on Moso bamboo in this study, examining the role of sap flow and culm photosynthesis to the culm CO_2_ efflux in leafless and leaved stages.

## 2 Materials and methods

### 2.1 Study site and bamboo culms

The experiment was conducted in the experimental garden (30°15’55” N, 119°42’47” E, 13 m asl) of Zhejiang A&F University in Hangzhou, located in southeast China. The climate of the study site belongs to the subtropical monsoon climate zone. The annual mean temperature was 17.6 ± 0.4°C, and the annual rainfall was 1579 ± 263 mm averaged from 2008 to 2017 (mean ± std; Mei et al., 2020). The studied bamboo stand produces a similar amount of bamboo shoots each year, different from the on-and-off-year bamboo forests. The upper canopy of bamboo culm that grew before 2015 was cut to avoid crushing by snow in winter, while the culms that developed after 2015 have whole canopies.

This study selected ten newly sprouted bamboo culms developed in the spring of 2018 and 2019, respectively. The selected culms had no visible damage from pests and diseases. Furthermore, they were in good condition, e.g., with an evenly rounded form, green shoot tips, brown hair on the cover of the culm sheaths, and no apparent indications of degradation. In April, the culms started falling off the sheaths at the breast height, developing leaves from the end of May, and finished expanding most of their new leaves by August (**Figure 1**). Therefore, the experiment was conducted from April to August, when newly sprouted culms were easily installed and monitored. More detailed bamboo phenology refers to another study conducted on the same site (Mei et al., 2020).

### 2.2 CO_2_ efflux measurement

**Figure 2:**
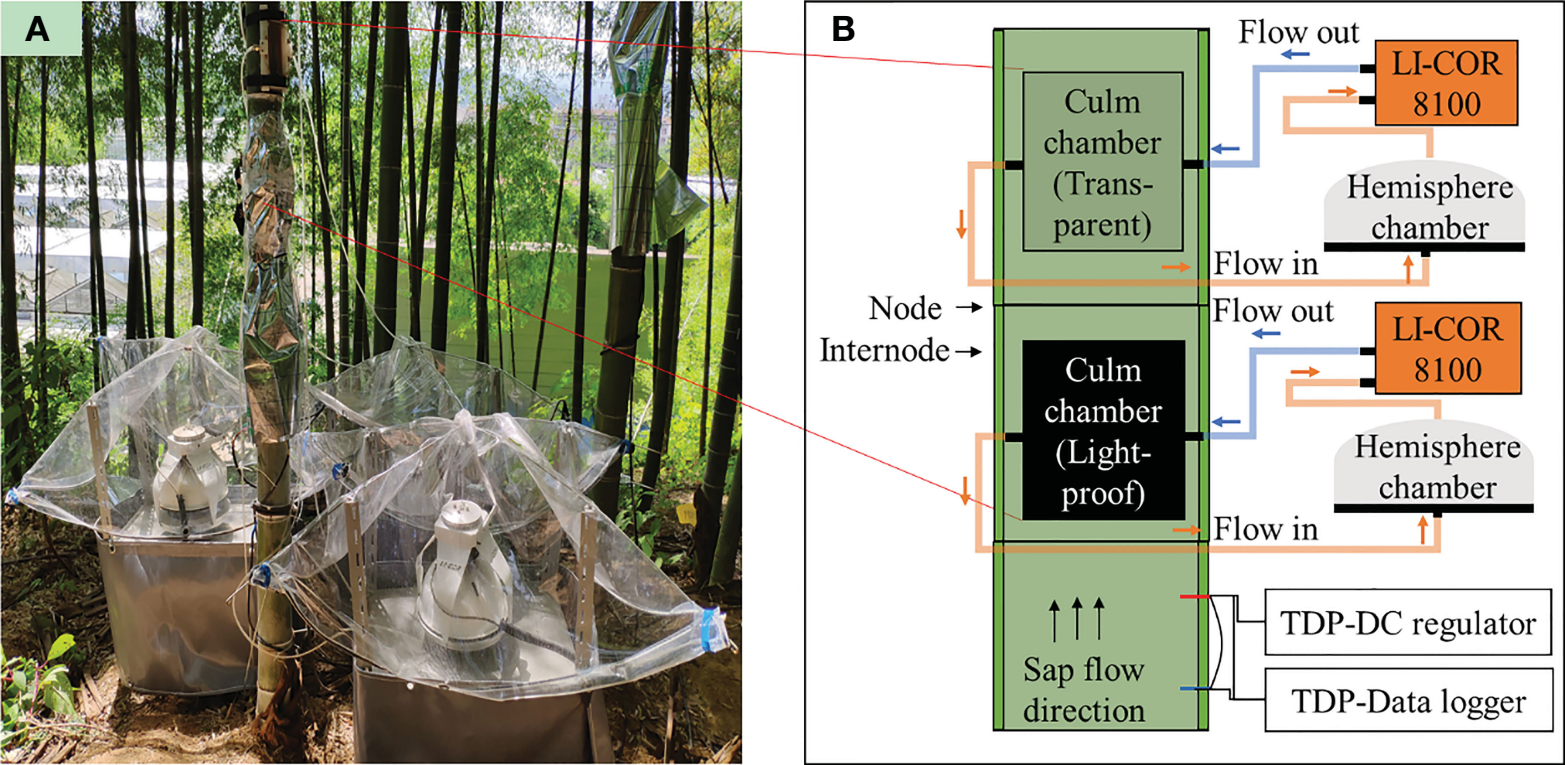
**(A)** Field installation and **(B)** schematic presentation of the equipment for monitoring CO_2_ efflux (*E*_s_, μmol m^−2^ s^−1^) in the field. The black and grey areas on the culm refer to light-proof and transparent culm chambers (double-chamber method). Each culm chamber was connected to an LI-Cor 8100 and a sealed hemisphere chamber. A pair of TDP was placed on an internode below the light-proof culm chamber. The sap flow direction was almost upward in the daytime.

*E*_s_ (μmol m^−2^ s^−1^) from the bamboo culm was measured with a self-sealing chamber designed following (Zhu et al., 2012). The chamber contains a cuvette covered with a 5*10 cm transparent PVC board and surrounded by 8 mm thick silicone foam to seal the gap between the chamber and the culm surface. Two plastic tubes with a row of holes were placed inside the cuvette on its left and right side. Two ends of each tube were connected to a T jointer that settled out of the cuvette, allowing airflow in and out. In application, the chamber was attached to the culm surface and fixed tightly with two belts to the bamboo culm (**Figure 2**).

The culm chamber was connected to a modified detector (LI-8100, Li-cor Inc., USA) to measure CO_2_ flux continuously. The LI-8100 contained an infrared CO_2_ analyzer and a hemisphere chamber designed. The hemisphere chamber was connected to the culm chamber with a tube in this study. Gas flowed into the hemisphere chamber from the culm chamber, mixed eventually, and sucked into the infrared CO_2_ analyzer by a build-in pump of the LI-8100. After measurement in the analyzer, the gas was pumped back into the culm chamber (**Figure 2**). When the measurement was running, the hemisphere chambers were closed, and the gas path of measurement became a closed-loop path. The chamber system was temporally closed for 5 min during measurements, and the air from the chamber was circulated using a pump from Li-cor 8100. The CO_2_ analyzer measured the CO_2_ concentration of the flow (ppm) and its temperature (°C). For each measurement, CO_2_ concentration started from an ambient value to a maximum accumulated concentration with a one-minute stepwise, given 30 s of a dead band, which excludes data from the beginning measurements and 50 cm^2^ of covered culm surface by the chamber, CO_2_ efflux (µmol m^−2^ s^−1^) can be derived with a linear model and recorded by the analyzer. After each measurement, the hemisphere chamber lifted, and air in both chambers was exchanged with the atmosphere for another 5 minutes.

Measurements were conducted from the beginning of April to the end of August in 2018 and 2019. A transparent and a light-proof chamber (double-chamber method) were installed separately on neighboring internodes for all studied culms (**Figure 2**). Two LI-8100s were equipped to both culm chambers and measured *E*_s_ simultaneously for three to five sunny days on a culm and then moved to another. Due to the rapid growth of freshly sprouted culms and limited days in each phenological stage, four culms in each year were measured in both 2018 and 2019.

### 2.3 Culm photosynthetic rate

*In-situ* respired CO_2_ (*R*_s_) was supposed to be released through four pathways (eq.1), including efflux from the culm surface (*E*_s_), stored at the position (*E*_I_), dissolved through sap flow (*E*_T_), and refixed by culm photosynthesis (*E*_p_), respectively.


(**eq.1**) $$R_{s}=E_{s}+E_{I}+E_{T}+E_{p}$$

Respiration is an enzyme-driven metabolism that is strongly influenced by temperature. Therefore, theoretical *R*_s_ (*R*_s_theory_, µmol m^−2^ s^−1^) can be derived with the following model:


(**eq.2**) $$R_{s\_theory}=\text{a}\times {\text{e}}^{\text{b}T}$$

where *T* is culm temperature (°C), which is assumed to be the same as the temperature of airflow measured by the infrared CO_2_ analyzer; a and b are parameters derived from nighttime *E*_s_ and *T*, during when *E*_T_ and *E*_p_ are assumed to be zero due to limited sap flow and photosynthetic photon flux density (PPFD). *E*_I_ is also supposed to be zero under balanced conditions during the night. Therefore, *R*_s_theory_ equals to *E*_s_ and parameters a and b could be derived from night-time observations (i.e., *E*_s_ and *T*).

*E*_s_ was always unequal to *in situ* respiration due to multiple partitioning pathways of respired CO_2_. The amount of the carbon loss of *R*_s_theory_ that was not released through the culm surface (*E*_s_) but through other pathways was named “missing efflux” in this study. A missing efflux rate (*E*_miss_) can be derived from *R*_s_theory_ − *E*_s_ for each observation step during a day.

To calculate culm photosynthesis, a transparent and a light-proof chamber were installed separately on neighboring internodes (**Figure 2**). Missing efflux rates for transparent (*E*_miss_) and light-proof (*E*_miss_) chambers were derived. Two equations can be derived from eq.1 for the transparent and light-proof chamber, respectively:


(**eq.3**) $$R_{s\_theory}=E_{s-tp}+E_{I-tp}+E_{T-tp}+E_{p-tp}$$

(**eq.4**) $$R_{s\_theory}=E_{s-lp}+E_{I-lp}+E_{T-lp}$$

As the two chambers were installed on the neighboring segments of a culm, we assumed that sap flow was similar and CO_2_ exchange to the storage of the hollow part was almost the same. Therefore, *E*_T−tp_ equals *E*_T−lp_, and *E*_I−tp_ equals *E*_I−lp_. Therefore, culm photosynthesis can be derived from eq.3 and eq.4.


(**eq.5**) $$\begin{array}{l} E_{p}=\left(R_{s\_theory}-E_{s-tp}\right)-\left(R_{s\_theory}-E_{s-lp}\right)\\ \;=E_{miss-tp}-E_{miss-lp} \end{array}$$

### 2.4 Sap flow measurement

Sap flux density on bamboo culms, where CO_2_ efflux was measured, was simultaneously monitored with a self-constructed thermal dissipation probe (TDP, Granier, 1987). A pair of TDP include one heating and one reference probes which are able to detect temperatures at the installation positions. The principle of TDP to measure sap flux is that the temperature will decrease as sap flux increases and bring heat around the heating probe away. The probes (1 cm in length) were modified from the 2-cm-length Granier-type probe (Granier, 1987) specifically for giant bamboo. The application and other specifications of self-build TDP refer to former studies on bamboo (Fang et al., 2019; Mei et al., 2020; Tong et al., 2021). When installing TDP on the culms, the heating and the reference probes were installed with a 10-cm vertical space at upper and lower positions. The upper heating probe was heated with 0.1 W power, and the lower reference probe was left unheated. The temperature difference between the two probes was recorded as voltage difference by a datalogger and multiplexers (CR1000, AM16/32, Campbell Inc., USA). Sap flux density (g m^−2^ s^−1^) was derived using the Moso bamboo-specific formula (Tong et al., 2021).


(**eq.6**) $$J_{s}=\text{μ}\times \;119\times {\left(\frac{Vmax}{V}-1\right)}^{1.231}$$

Where *J*_s_ is sap flux density (g m^−2^ s^−1^), *V*_max_ is the maximum output voltage in a day, which usually appears at night when sap flow is almost zero. *v* is a mean output voltage every 10 minutes. Finally, μ is the age-specific parameter for Moso bamboo (Tong et al., 2021).

### 2.5 Micrometeorological observations

Two micrometeorological stations were set up above and below the bamboo canopy. The above-canopy station was located about 10 m from the stand in an open field. Photosynthetic Photon flux density (PPFD, LI190R, Campbell Scientific, USA), air temperature, and humidity (HMP155A, Campbell Inc., USA) were measured in the above canopy station. The below-canopy station was placed in the stand to monitor radiation below the canopy (LI190R, Campbell Scientific, USA) and soil moisture (CS616, Campbell Inc., USA). Three soil moisture probes were placed at the study site. Meteorological data were collected by dataloggers (CR1000, Campbell Inc., USA).

### 2.6 Data analysis and statistics

Daily patterns of hourly *E*_s_, *E*_miss_ were plotted for culms in transparent and light-proof chambers, and so was hourly *E*_p_ for culms in transparent chambers in the leafless and leaved stage of the newly sprouted culms.

The difference in stem surface temperature, measured efflux, and theoretical respired efflux between transparent and light-proof chambers were examined with Student’s t-test (parametric method) and Signed Rank methods (nonparametric method) if data was normal and non-normal distribution, respectively.

Daily accumulated *E*_miss_ and *E*_p_ were examined to determine if they had linear relationships with daily accumulated sap flux density and environmental variables (radiation above and below canopy, air temperature, air humidity, soil moisture). And significant regressions were plotted.

Furtherly, stepwise linear multiple regression models predicting *E*_s_, *E*_miss_, and *E*_p_ with environmental factors were conducted to explore the comparative implications of varying factors. Only variables entered in the models were kept for analysis.

Percentages of daily *E*_s_, *E*_miss_ to *R*_s_theory_ were calculated for culms in transparent and light-proof chambers. Further, the daily *E*_p_ to *R*_s_theory_ ratio was calculated for transparent chamber culms. As *E*_miss_ and sap flow had significant positive relationships, as examined above, we supposed the regression *R* ^2^ between them could be a proxy of *E*_T_ to *E*_miss_, which was furtherly multiplied by the Percentage of *E*_miss_ to *R*_s_theory_ to obtain the Percentage of *E*_T_ to *R*_s_theory_. At last, the Percentage of *E*_I_ to *R*_s_theory_ was calculated by subtracting the above three parts from 1.

Except for **Figures 1**, **2**, all the other figures and analyses were performed with SAS 9.4 (SAS Institute Inc., Cary, NC, USA).

## 3 Results

### 3.1 CO_2_ efflux (*E*_s_) of freshly sprouted Moso bamboo

**Figure 3:**
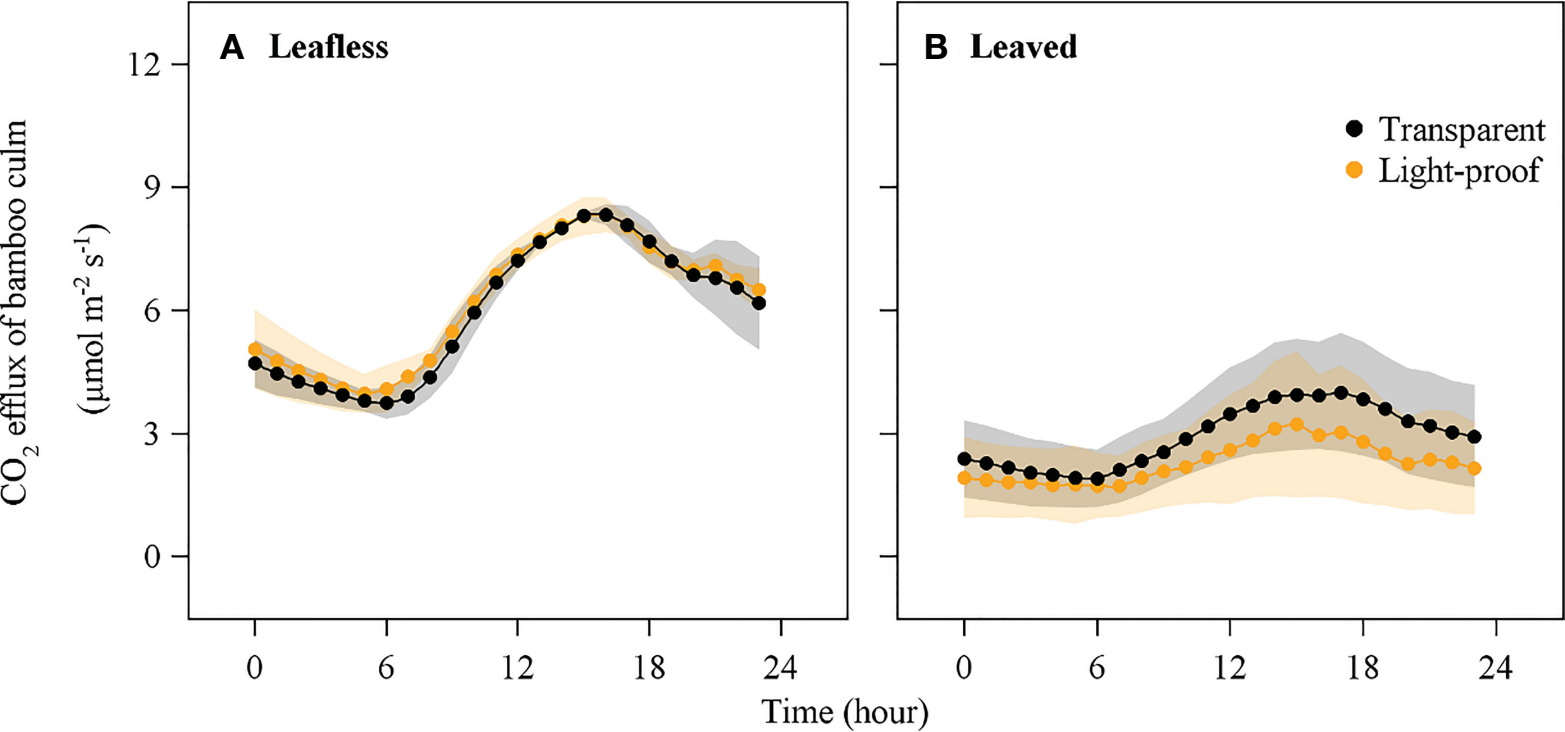
CO_2_ efflux of bamboo culm (*E*_s_) in the transparent and light-proof chambers in leafless **(A)** and leaved **(B)** stages (mean ± std, n = 8).

On freshly sprouted Moso bamboo culms at both phenological stages (leafless and leaved), *E*_s_ showed a similar daily pattern, i.e., higher value in the daytime and lower value in the nighttime (**Figure 3**). *E*_s_ increased rapidly at around 6:00 in the morning, reached its maximum value at 14:00–15:00, and decreased afterward.

There was no significant difference in *E*_s_ between transparent and light-proof chambers in the leafless stage (*P* < 0.05), while *E*_s_ in transparent chambers were significantly higher than that in light-proof chambers in the leaved stage (*P* < 0.05; **Table 1**). In contrast, in both types of chambers, *E*_s_ was significantly higher in the leafless stage than in the leaved stage (*P* < 0.05). The maximum value of *E*_s_ in a day was 8.5 ± 0.9 μmol m^−2^ s^−1^ in the leafless stage and 3.6 ± 1.5 μmol m^−2^ s^−1^ in the leaved stage. Mean *E*_s_ was 4.7 ± 1.1 μmol m^−2^ s^−1^ in the leafless stage and 2.3 ± 0.9 μmol m^−2^ s^−1^ in the leaved stage (**Figure 3**).

**Table 1:** The difference in measured efflux (dif_*E*_s_), stem surface temperature (dif_ temperature), and theoretical respired efflux (dif_*R*_s_theory_) between transparent and light-proof chambers.

VarName	Time	Stage	Mean_std	Significance	Test method	P Value
dif_*E*_s_	nighttime	leafless	0.52(4.39)	-	Signed Rank	0.90
		leaved	2.69(2.35)	**	Student's t	<.01
	whole_day	leafless	2.52(9.27)	-	Signed Rank	0.63
		leaved	6.51(5.31)	**	Student's t	<.01
dif_ temperature	nighttime	leafless	0.05(0.22)	-	Signed Rank	0.39
		leaved	0.09(0.22)	**	Student's t	0.03
	whole_day	leafless	0.11(0.21)	-	Student's t	0.08
		leaved	0.11(0.18)	**	Student's t	<.01
dif_ *R*_s_theory_	nighttime	leafless	0.55(4.38)	-	Signed Rank	1.00
		leaved	2.71(2.37)	**	Student's t	<.01
	whole_day	leafless	6.53(12.15)	-	Student's t	0.07
		leaved	10.12(6.98)	**	Signed Rank	<.01

Double asterisks indicate that *P*-values for the significance test is < 0.05.

### 3.2 Impact factors of *E*_s_

**Figure 4:**
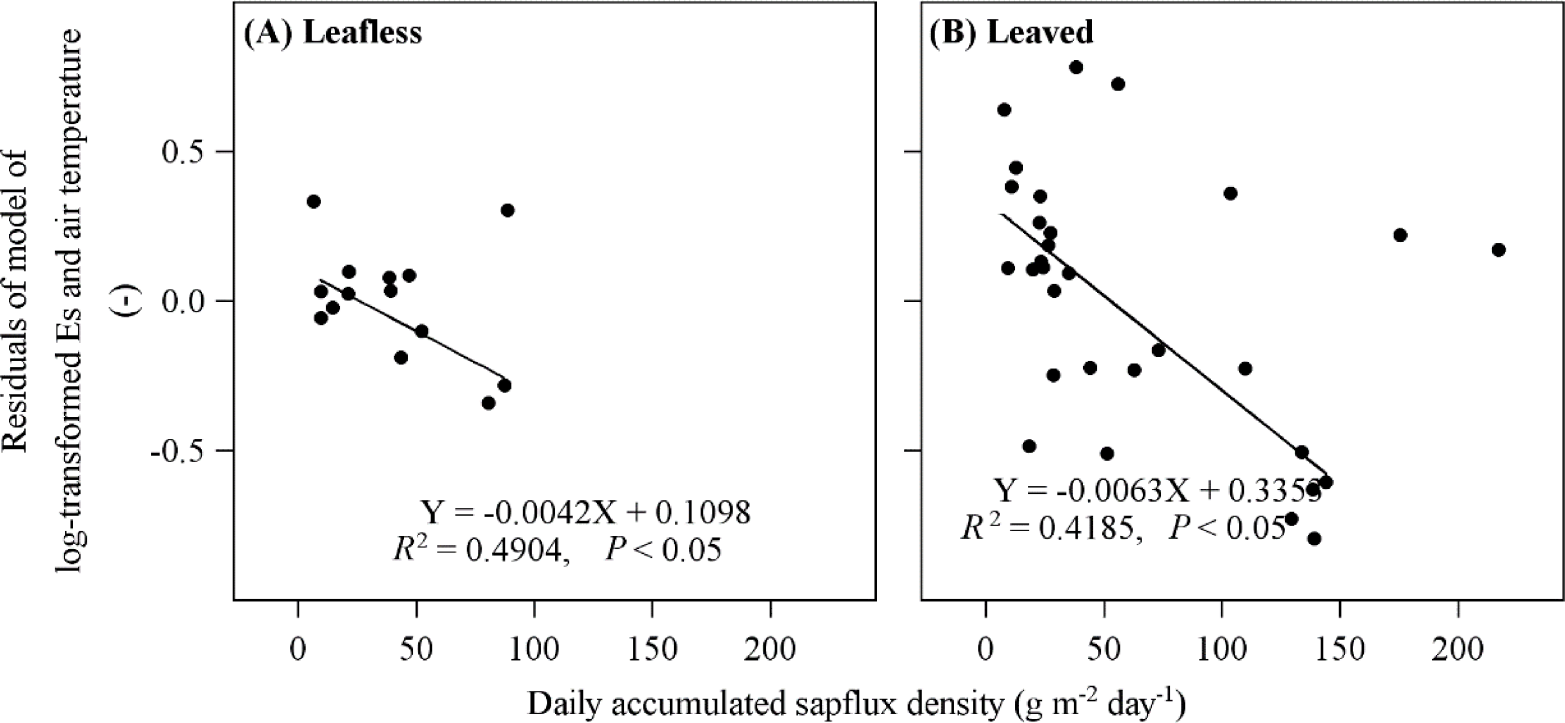
Relationship between daily accumulated sap flux density and residuals of the log-transformed stem-surface CO_2_ efflux (*E*_s_) model and air temperature in leafless **(A)** and leaved **(B)** stages of the newly sprouted culms. One and two abnormal points were excluded from the regressions in leafless **(A)** and leaved **(B)** stages, respectively.

This study conducted a stepwise multiple regression to explore the comprehensive impacts of the varying environmental factors on *E*_s_ in transparent chambers (**Table 2**). For both leafless and leaved stages, three variables (sap flux density, below-canopy radiation, and temperature) entered the models (*P* < 0.01 and *R* ^2^ = 0.7). However, the leading impact factor differed in the two stages, i.e., sap flux (model variation explained = 67.6%) for the leafless stage and below-canopy radiation (model variation explained = 78.6%) for the leaved stage. In both stages, sap flux density and below-canopy radiation exerted significant negative and positive effects on *E*_s_. In contrast, below-canopy temperature shifted its impact on *E*_s_ from positive to negative when the newly sprouted culms changed their status from leafless to leaved. To find more relationships between sap flux density and *E*_s_, we calculated the residuals of the model of log-transformed *E*_s_ and air temperature in the leafless and leaved stages to eliminate the effect of temperature. Then sap flux density significantly negatively affected *E*_s_ (**Figure 4**), respectively.

**Table 2:** Stepwise linear multiple regression model predicting *E*_s_ with environmental factors.

Stage	Prob F	R^2^	Variable Entered	Parameter Estimate	Partial R-Square	var_explained
leafless	<.01	0.72	Sap flux density	−0.19	0.48	67.6%
			Below-canopy radiation	1.61	0.15	21.0%
			Below-canopy temperature	1.07	0.08	11.4%
			Intercept	14.36	.	.
leaved	<.01	0.71	Below-canopy radiation	2.81	0.56	78.7%
			Below-canopy temperature	1.38	0.11	15.9%
			Sap flux density	−0.05	0.04	5.4%
			Intercept	50.39	.	.

### 3.3 Culm photosynthesis

**Figure 5:**
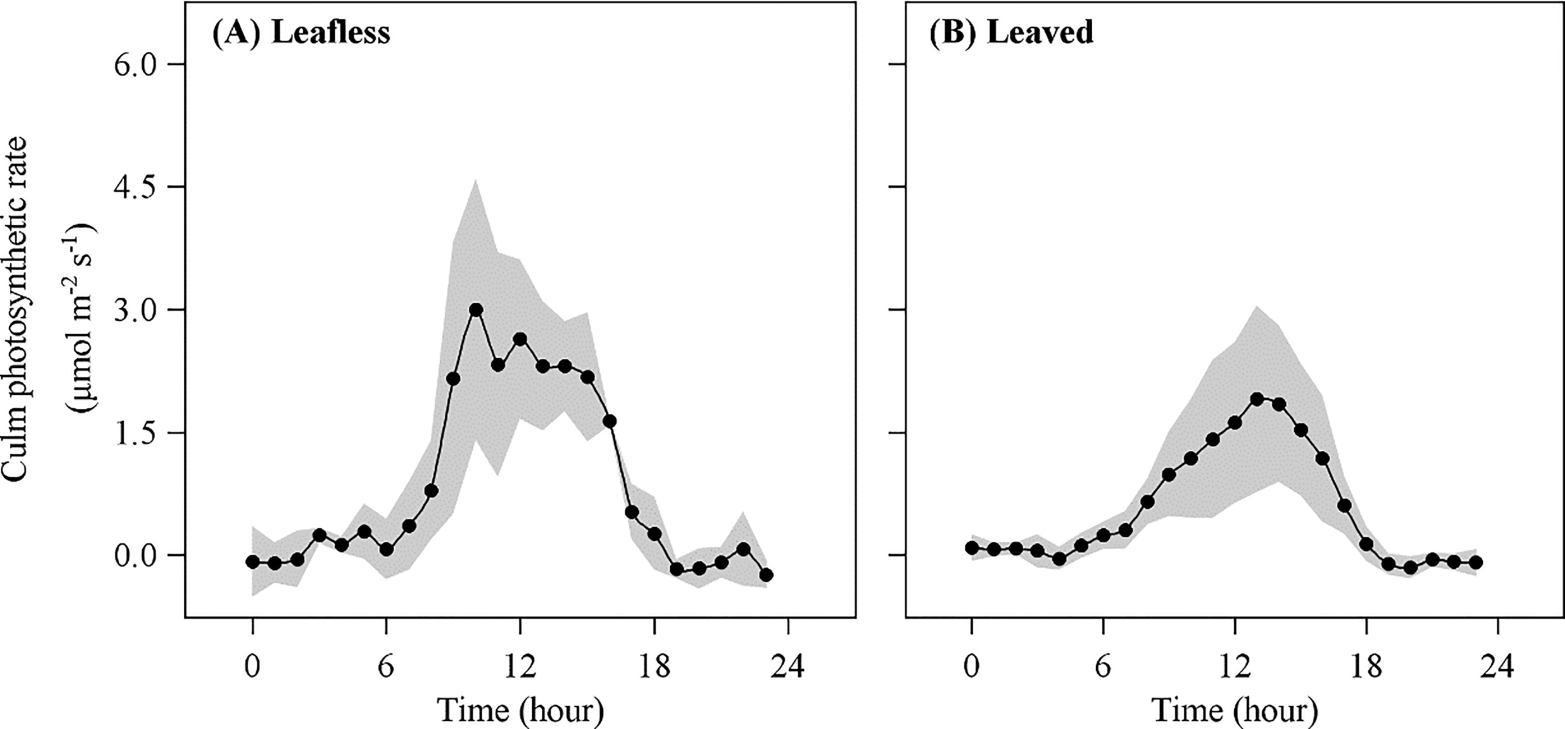
Culm photosynthetic rate (*E*_p_) in leafless **(A)** and leaved **(B)** stages of the newly sprouted culms.

**Figure 6:**
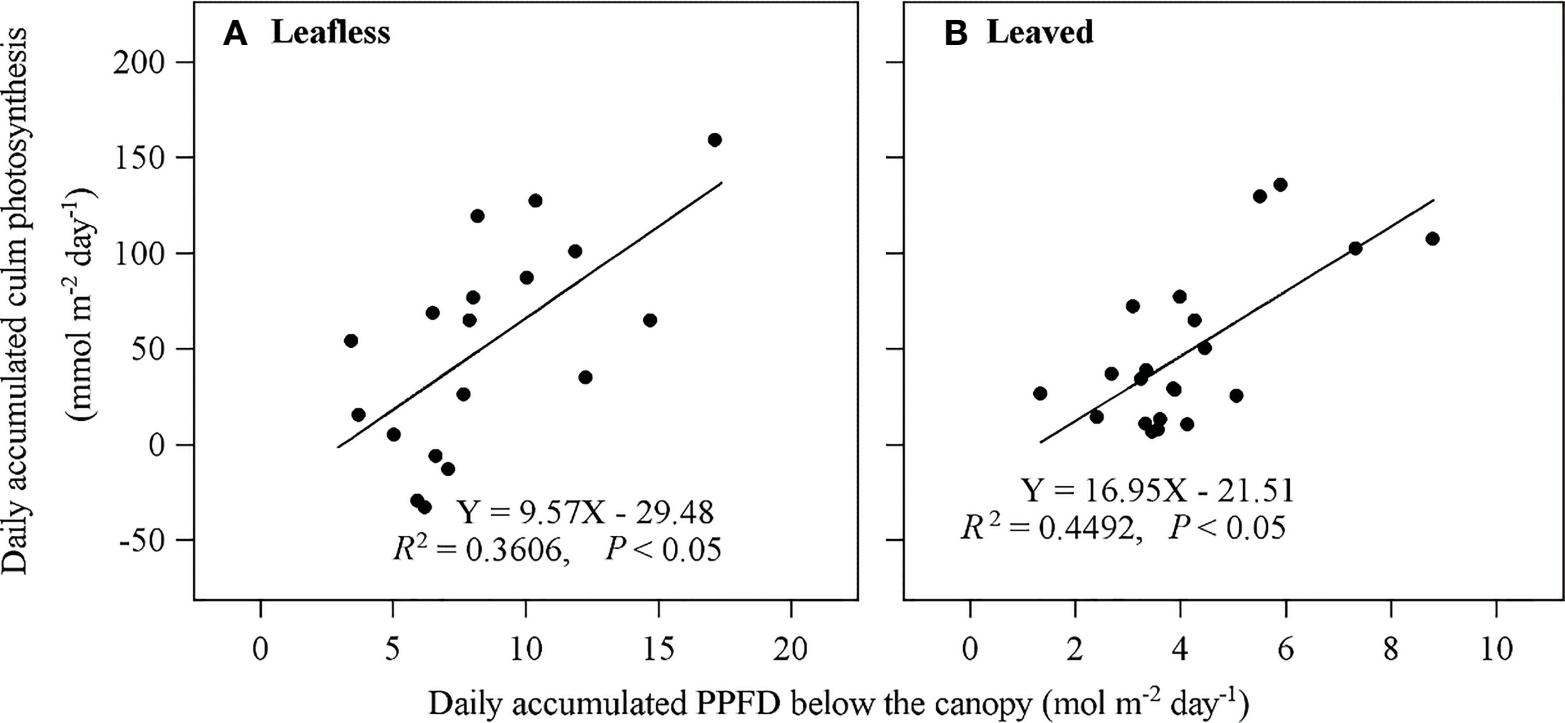
Relationship between daily accumulated culm photosynthesis (*E*_p_) and daily accumulated radiation (PPFD) below the canopy in leafless **(A)** and leaved **(B)** stages the newly sprouted culms.

In transparent chambers, *E*_P_ presented a typical single-peak pattern with a maximum value around mid-day and a lowest at night (**Figure 5**). Daily accumulated *E*_p_ was 81.74 ± 42.40 mmol m^−2^ day^−1^ and 39.05 ± 6.65 mmol m^−2^ day^−1^ in leafless and leaved stages, respectively, which meant ~9.0% (with a maximum value of 33.4%) and 13% (with a maximum value of 40.4%) of *R*_s_, respectively. *E*_p_ contributed half of the daily accumulated *E*_miss_ (**Table 3**). In addition, daily accumulated *E*_p_ has a significant correlation (*P* < 0.05) with daily accumulated below-canopy radiation in both stages (**Figure 6**).

**Table 3:** Partitioning percentages of culm-respired CO_2_ efflux flowing to the four pathways, i.e., surface (*E*_s_), photosynthesis (*E*_p_), sap flow (*E*_T_), and internal storage (*E*_I_).

Chamber type	Stage	Percentage of *E*_s_ to *R*_s_ (%)	Percentage of *E*__missing_ to *R*_s_ (%)	Percentage of *E*_p_ to *R*_s_ (%)	Percentage of *E*_T_ to *R*_s_ (%)	Percentage of *E*_I_ to *R*_s_ (%)
Transparent	leafless	82±9	18±09	9±11		
	leaved	80 ±12	20±12	13±15		
Light-proof	leafless	91±14	9±14		2 (-)	7 (-)
	leaved	89±13	11±13		2 (-)	9 (-)

### 3.4 Missing efflux of *in situ* respired CO_2_ and its impact factors

**Figure 7:**
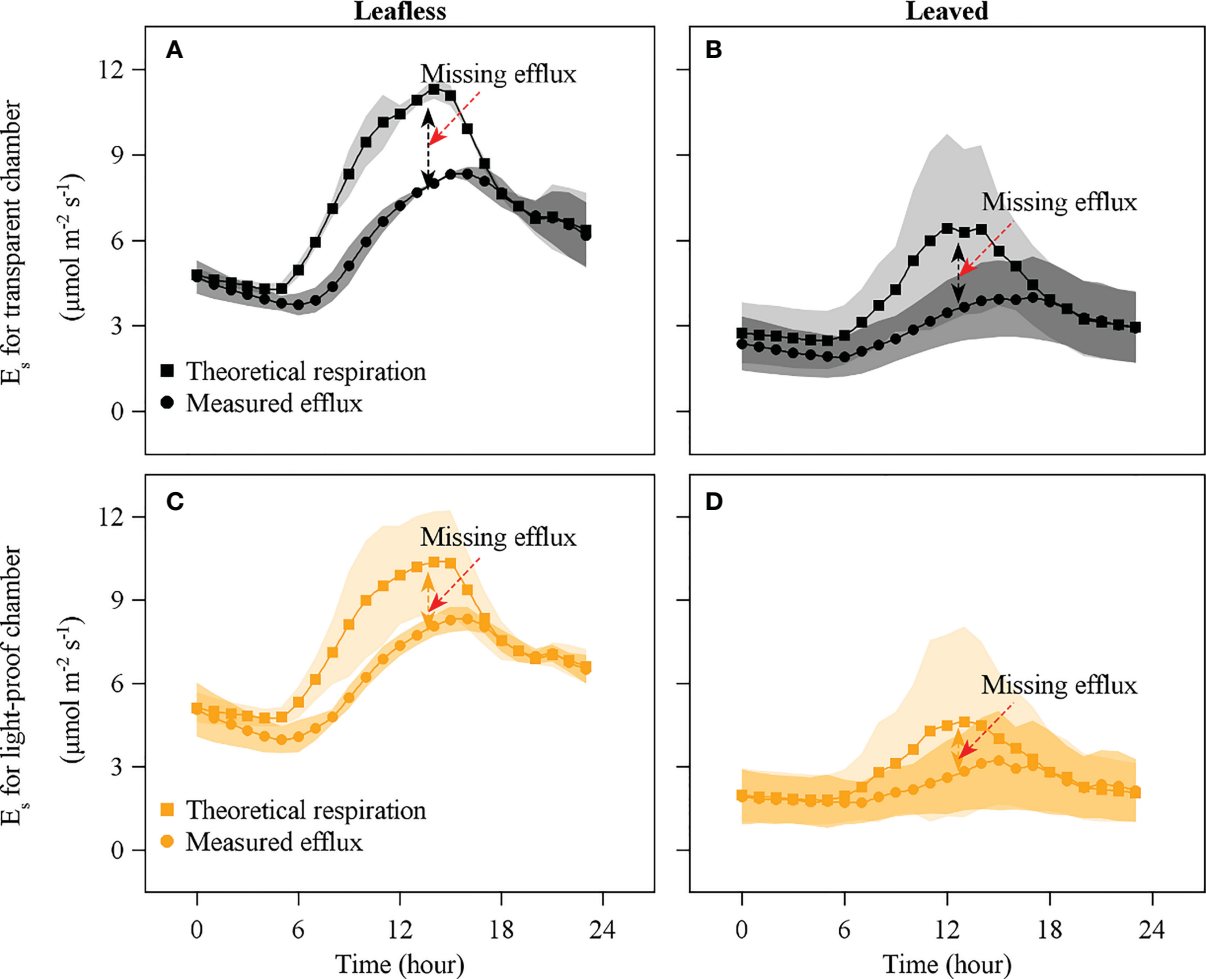
Comparison of theoretical respiration (squares) and measured efflux (dots) with transparent (black) and light-proof chambers (orange) in leafless **(A)**, **(C)** and leaved **(B)**, **(D)** stages. Note that E_s_ is the CO_2_ efflux released from the culm surface, and missing efflux is the difference between the theoretical respiration and *E*_s_.

**Figure 8:**
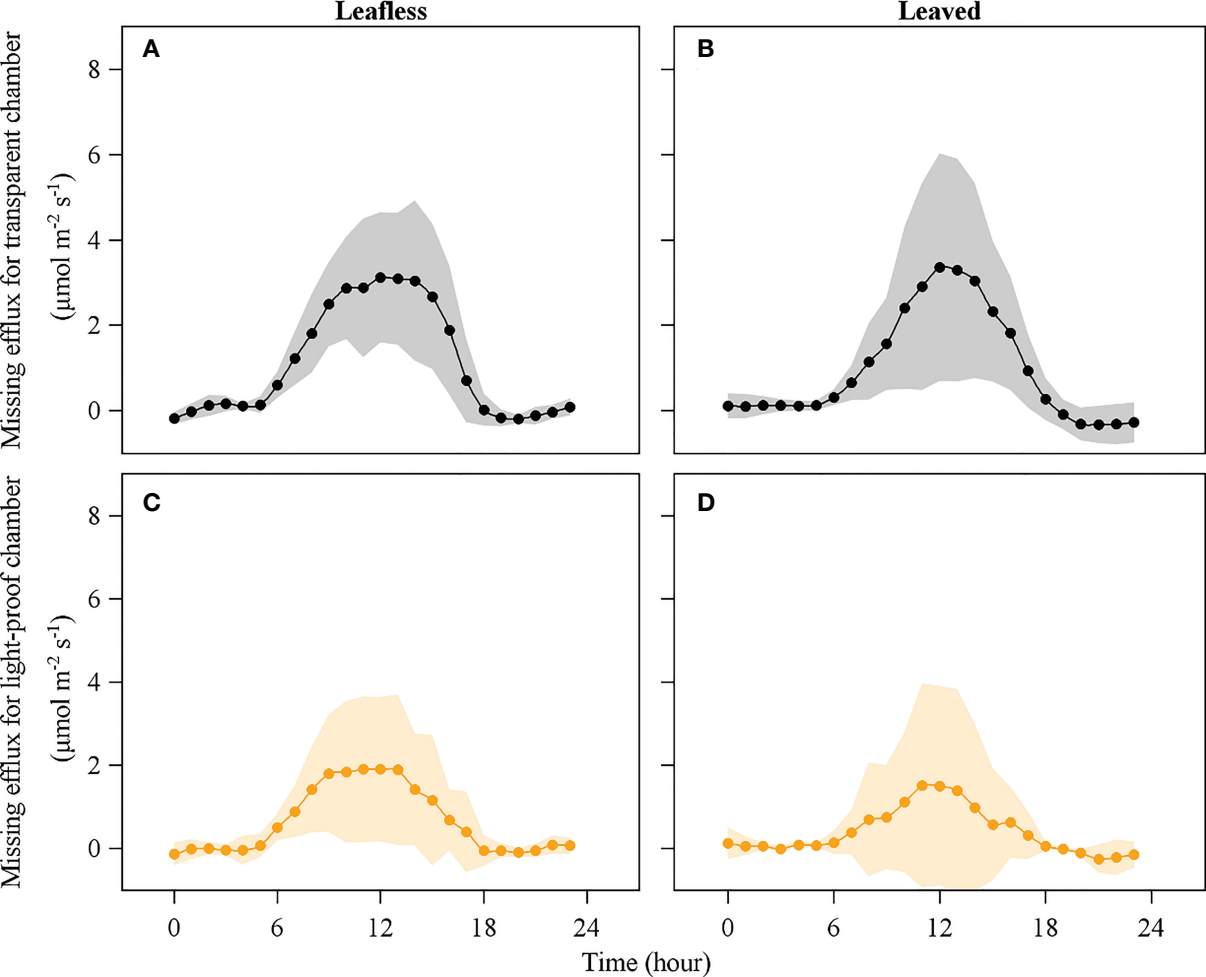
Missing efflux of culm respired CO_2_ in leafless **(A)**, **(C)** and leaved **(B)**, **(D)** stages for transparent and light-proof chambers. Note that missing efflux is the difference between the theoretical culm respiration and the surface-released CO_2_ efflux.

An apparent *E*_miss_ was observed during daytime in the leafless and leaved stages for both the transparent and light-proof chambers (**Figures 7**, **8**). There was no significant difference in daily accumulated *E*_miss_ between leafless and leaved stages for each type of chamber (*P* > 0.05). In contrast, a significant difference was observed between the transparent and light-proof chambers for both stages (*P* < 0.05). In both stages, daily accumulated *E*_miss_ accounts for ~10% and 20% of *R*_s_ in transparent and light-proof chambers, respectively (**Table 3**).

**Figure 9:**
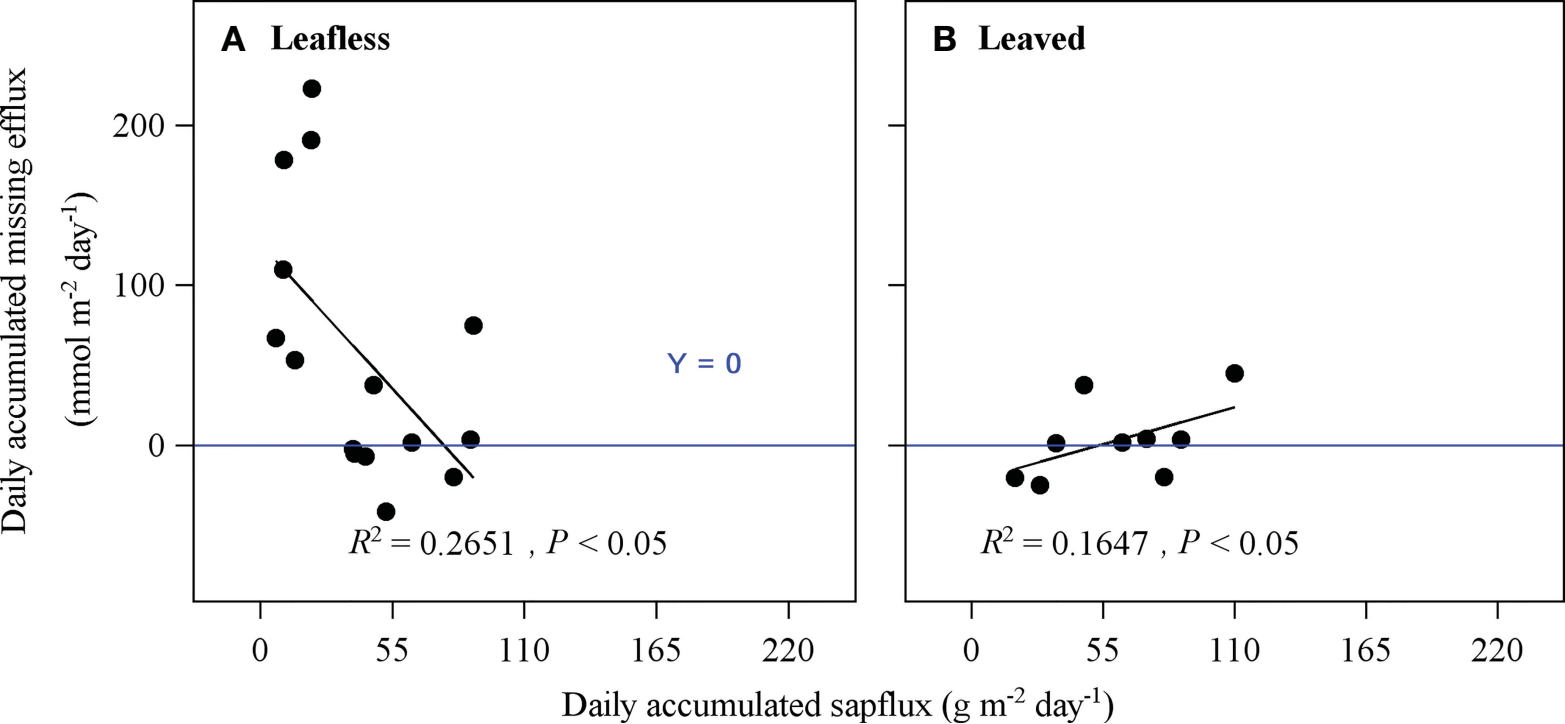
Relationships of daily accumulated sap flux and daily accumulated missing efflux of the light-proof chamber (i.e., exclude culm photosynthesis) in leafless **(A)** and leaved **(B)** stages. Note that missing efflux is the difference between the theoretical culm respiration and the surface-released CO_2_ efflux.

Daily accumulated *E*_miss_ had significantly negative (*R* ^2^ = 0.27) and positive (*R* ^2^ = 0.16) relationships with daily accumulated sap flux in leafless and leaved stages, respectively (*P* < 0.05; **Figure 9**), which may indirectly indicate a similar contribution (~27 and 16%) of *E*_T_ to *E*_miss_. In this case, the results may furtherly mean that *E*_T_ could merely account for ~2% of *R*_s_. As *E*_miss_ consisted of *E*_T_ and *E*_I_, the contribution of *E*_I_ to *R*_s_ could be ~7–9% by subtracting *E*_T_ from *E*_miss_ (**Table 3**).

In stepwise multiple regression predicting *E*_miss_, two variables entered the models for both leafless (*P* < 0.01; *R* ^2^ = 0.58) and leaved stages (*P* < 0.01; *R* ^2^ = 0.32; **Table 4**). Below-canopy radiation was the most influential variable for both stages, explaining 61% and 58% of the model variation in leafless and leaved stages, respectively. In contrast, the second most significant variable was different, i.e., below-canopy temperature and sap flux for leafless and leaved stages, respectively.

**Table 4:** Stepwise linear multiple regression model predicting *E*__miss_ with environmental factors.

Stage	ProbF	R^2^	Variable Entered	Parameter Estimate	Partial R-Square	Variance Explained
Leafless	<.01	0.58	Below-Canopy Radiation	1.27	0.35	60.8%
			Below-Canopy Temperature	1.09	0.23	39.2%
			Intercept	−24.6	.	.
Leaved	<.01	0.32	Below-Canopy Radiation	0.68	0.19	57.6%
			Sap flux density	0.04	0.14	42.4%
			Intercept	0.45	.	.

## 4 Discussion

### 4.1 CO_2_ efflux of newly sprouted Moso bamboo culm between leafless and leaved stages

Compared to the averaged *E*_s_ (2.04 ± 2.03 μmol m^−2^ s^−1^) of all the studied tree/bamboo species in our synthesized data pool (**Figure S1**), the newly sprouted Moso bamboo culms released a much higher and similar *E*_s_ in leafless and leaved stages, respectively. Furtherly, compared with *E*_s_ in the previous studies on bamboo (**Table 5**), *E*_s_ in the leaved stage in this study was similar to the value reported on 1–2-year-old Moso bamboo in China (2.3 μmol m^−2^ s^−1^; Xiao et al., 2010) and 4-month-old ones in Japan (1.9 ± 0.46 μmol m^−2^ s^−1^; Uchida et al., 2022) but much lower than *E*_s_ of 6-month-old *Bambusa vulgaris* (6.9 μmol m^−2^ s^−1^; Zachariah et al., 2016). *E*_s_ of Moso bamboo measured *in situ* showed a decreasing trend with increasing ages (**Table 5**, and Xiao et al., 2010; Uchida et al., 2022), almost coinciding with findings based on harvested culm segments from another previous study (Isagi et al., 1997).

**Table 5:** Comparison of bamboo culm efflux of this study and bamboos in former studies, and contribution of culm photosynthesis to theoretical *in situ* respiration.

Bamboo species	Culm age	Daily mean *E*_s_ (μmol m^−2^ s^−1^)	Measurement time	Reference
Moso bamboo	1–2 months	4.7±1.1	Whole day	This study
	3–4 months	2.3±0.9		This study
	12–24 months	2.3 (-)		Xiao et al., 2010
	~4 months	1.9±0.5	Before dawn	Uchida et al., 2022
	>24 months	0.17±0.09		Uchida et al., 2022
*Bambusa vulgaris*	6 months	6.9 (-)		Zachariah et al., 2016
	12 months	1.8 (-)		Zachariah et al., 2016

The decreasing trend of *E*_s_ by aging could be attributed to potentially reduced maintenance respiration in the cytoplasm, which could be squeezed by the thicking cell wall of parenchyma (Uchida et al., 2022) and fiber (Huang et al., 2015). In the leafless stage, the current-year newly sprouted culms elongated their internodes with increasing cell length and the number of parenchymas and high lignin and cellulose content (Chen et al., 2022), which meant active growth respiration consuming a mass of carbohydrates, e.g., starch (Uchida et al., 2022). The rule that growth stimulated higher *E*_s_ was also supported in trees in several studies (Ryan, 1990; Maier, 2001; Vose and Ryan, 2002; Lavigne et al., 2004). In this study, the newly sprouted Moso bamboo culms have about twice as high *E*_s_ in leafless stages than in leaved stages (**Figure 3**). If taking the after-leaved *E*_s_ as a reference for maintenance respiration, growth respiration during the leafless stage accounted for more than 50% of the total respiration. Therefore, growth could contribute mainly to the higher *E*_s_ of newly sprouted bamboo culm in the leafless stage.

For both leafless and leaved stages, three variables (sap flux density, below-canopy radiation, and temperature) entered the models for predicting *E*_s_ (*P* < 0.01 and *R* ^2^ = 0.7; **Table 2**). However, the leading impact factor differed in the two stages, i.e., sap flow for the leafless stage and below-canopy radiation for the leaved stage **(** **Table 2**). In both stages, sap flux density had significantly negative correlations with *E*_s_, which is consistent with some former studies (McGuire and Teskey, 2004; Bowman et al., 2005). Bowman et al. (2005) found that sap flow could interpret variables of *E*_s_ among different heights, directions, and individuals. Direct monitoring of dissolved CO_2_ concentration in sap flow for *Fagus grandifolia*, *Liquidambar styraciflua*, and *Platanus occidentalis* showed 13–71% of the respired CO_2_ was transported through sap flow and hence positively correlated with sap flow (McGuire and Teskey, 2004). A considerable increase in sap flow for freshly sprouted Moso bamboo in the same site was observed (Mei et al., 2020), which may take away the *in situ* respired CO_2_ and reduce *E*_s_ after leaved. However, sap flow can only explain 5.4% of the variation of *E*_s_ in the leaved stage (**Table 2**). Such a result does not fit our first assumption that sap might take away more CO_2_ efflux and negatively influence *E*_s_ in the leaved stage more than in the leafless stage.

### 4.2 Roles of culm photosynthesis on carbon partitioning of *in situ* respired CO_2_

In this study, culm photosynthesis was assumed to equal the difference value of *E*_miss_ between transparent and light-proof chambers. The two-chamber design attempted to avoid errors in calculating culm photosynthesis that was introduced by CO_2_ partitioning through other pathways, e.g., sap flow, compared with the single-chamber design reported by Tarvainen et al. (Tarvainen et al., 2018). The single-chamber design works on one assumption that carbon partitioning through sap flow was ignorable (Tarvainen et al., 2018). However, some former studies did not support this assumption (McGuire and Teskey, 2002; Saveyn et al., 2008). Therefore, the two-chamber design applied in this study was expected to improve the estimation accuracy in culm photosynthesis.

As one of the partitioning pathways for *in situ* respired CO_2_, culm photosynthesis was supposed to reduce *E*_s_ (Pfanz et al., 2002; Wittmann et al., 2006). The above assumption was indicated by observing a smaller *E*_s_ with culm photosynthesis in this study (**Table S2**). Due to the high radiation dependence of photosynthesis and reduced radiation below the canopy with expanded leaves for a specific freshly sprouted culm, culm photosynthesis was assumed to reduce in the leaved stage. The above assumption was also confirmed in this study, i.e., a significantly reduced PPFD below the canopy after June (7.05 ± 4.72 *vs*. 4.37 ± 1.97 mol m^−2^ day^−1^ in the leafless and leaved stages, respectively), along with a significantly reduced culm photosynthesis in the leaved stage (**Figures 5**, **6**). Higher culm photosynthesis in leafless stages helps recycle more carbon loss due to respiration for freshly sprouted culms. However, *E*_p_ has a relative contribution (~10%) to the partitioning of *R*_s_ in both stages, which does not fit our hypothesis that its effect may be reduced in the leaved stage due to the increased sap flow.

To explore whether *E*_s_ of this study falls into a reasonable range, we collected *E*_s_ of 195 tree species in different climate zones from articles published before February 13, 2020 (**Figure S1**). As a result, *E*_s_ of Moso bamboo in this study falls in a promising position close to the respiration-temperature regression line of subtropical tree species (**Figure S1**). It was predicted to have a lower *E*_s_ on species with culm photosynthesis. The green bamboo culm was proved to have photosynthesis (Liu et al., 2013; Wang et al., 2013) and hence lower *E*_s_. Almost all the studied bamboo *E*_s_ falls close to/below the respiration-temperature regression line based on the collected worldwide dataset (**Figure S1**), which was in line with the above assumption, except for one case study on *Bambusa vulgaris* conducted in the tropical zone.

In this study, the daily accumulated *E*_p_ was significantly positively correlated with daily accumulated PPFD below the canopy (**Figure 6**), agreeing with previous expectations. As one of the partitioning pathways of respired CO_2_, culm photosynthesis could recycle about 9–13% of the *in situ* respired CO_2_ (**Table 3**). Culm photosynthesis could be essential to trees originally located in high-latitude areas worldwide. By synthesizing *E*_s_ data from articles published before February 13, 2020, we found a significant relationship between *E*_s_ and temperature in each climate zone (**Figure S1**). From the dataset, we observed a lower *E*_s_ on tree species with non-culm photosynthesis (temperate zone 2.64 μmol m^−2^ s^−1^; subtropical zone 2.24 μmol m^−2^ s^−1^) compared to tree species with culm photosynthesis (temperate zone 3.18 μmol m^−2^ s^−1^; subtropical zone 4.15 μmol m^−2^ s^−1^). As a photo-induced physiological process, culm photosynthesis could be more likely to appear in forests with fewer-layer canopies than multi-layer canopies. With increasing latitude, forest composition and structure tend to be simpler, allowing more light to penetrate the stand canopy. Although Moso bamboo is considered an evergreen species, it renews leaves in early spring when explosive growth occurs every two years (Mei et al., 2020), leading to higher radiation below the canopy during this stage (**Figure 6B**). The spring leafing phenology seems to favor culm photosynthesis and the growth of young bamboo culms (Mei et al., 2020).

### 4.3 Missing efflux and partitioning of *in situ* respired CO_2_

An apparent *E*_miss_ was observed during daytime in the leafless and leaved stages for both the transparent and light-proof chambers (**Figures 7**, **8**, and **Table 3**). *E*_p_ accounted for 50% of *E*_miss_, equaling a partitioning of 10% of *R*_s_. Therefore, the left ~10% of *R*_s_ might be partitioned to the pathways of sap flow and internal storage, i.e., *E*_T_ and *E*_I_. However, the experimental design in this study can not precisely distinguish *E*_T_ and *E*_I_ with direct measurement, which meant either *E*_T_ or *E*_I_ might account for 10% of *R*_s_ at most in extreme cases. Alternatively, we roughly estimated the *E*_T_ could merely account for ~2% of *R*_s_ *via* the correlations between *E*_miss_ and sap flow (**Figure 9**), thus deriving the percentage of *E*_I_ (~8%).

In stepwise multiple regression predicting *E*_miss_, two variables entered the models for both leafless and leaved stages (**Table 4**). Below-canopy radiation was the most influential variable for both stages. The result indirectly confirmed the partitioning pathway of culm photosynthesis, considering the positive relations between radiation and photosynthesis.

For a newly sprouted culm with a DBH of 10.77 cm and a height of 13.41 m, its estimated aboveground biomass was about 12.105 kg calculated with an allometric equation ( $\text{Aboveground Biomass}=0.712\times DBH^{1.477}$ , Zhang et al., 2016). Based on daily accumulated *E*_P_ and growth rates (48.99 cm d^–1^) in leafless stages (Chen et al., 2022), we found the total fixed carbon from culm photosynthesis accounted for 2.40% of the aboveground biomass of bamboo in leafless stages.

Consistent with some previous studies (McGuire and Teskey, 2004; Teskey et al., 2007; Aubrey and Teskey, 2009), daily accumulated sap flux density was proved to have a significant negative effect on *E*_miss_ (**Figure 9**). The above correlation may confirm the carbon partitioning of *in situ* respired CO_2_ taken by sap flow. In addition, a significant positive relationship between sap flux density and *E*_miss_ was found in the leaved stage in this study (**Figure 9B**), which also implied a possible carbon partitioning of *in situ* respired CO_2_ through sap flow. However, as mentioned above, sap flux density can only explain 5.4% of the variation of *E*_s_ in the leaved stage (**Table 2**).

## 5 Conclusion

The applied double-chamber method was used to estimate culm photosynthesis in newly sprouted Moso bamboo, which could provide a powerful way to test the mass balance framework (MBF) on stem respiration (*R*_s_). By measuring and analyzing culm photosynthesis, sap flux, and micrometeorological factors above and below the canopy, we found 80% of *in situ R*_s_ were released through the culm surface of Moso bamboo. In comparison, culm photosynthesis and sap flux reallocated approximately 10% and 2% of *in situ R*_s_. Despite higher culm photosynthesis in the leafless stage and a higher sap flow in the leaved stage, the carbon partitioning pathways indicated by MBF have no significant difference between the two phenological stages. Considering the dynamic change of the partitioning components of *in situ R*_s_ and the handleability of the developed method on bamboo culm, we think bamboo is a good choice to explore the MBF.

## Data availability statement

The original contributions presented in the study are included in the article/**Supplementary Material**. Further inquiries can be directed to the corresponding authors.

## Author contributions

TM, GZ and CY planned and designed the research; CY, KH, YZ and TM performed experiments, and conducted fieldwork; CY, QZ and DF analyzed data; and CY, QZ, KH, DF, DH, HD, YS, FB, TM, GZ wrote or revised the manuscript. CY and QZ contributed equally. All authors contributed to the article and approved the submitted version.
