# Supplementary material for: Partitioning of respired CO2 in newly sprouted Moso bamboo culms
Source: Front Plant Sci. 2023 Apr 20;14:1154232. doi: 10.3389/fpls.2023.1154232 (PMC10158728; doi:10.3389/fpls.2023.1154232)
Supplement: Supplementary file 1 [file DataSheet_1.docx]

**Global-scale synthesis for stem respiration and stem photosynthesis**

To explore the effects of temperature and stem photosynthesis on stem *E*_s_ across tree species, particularly to compare the difference between bamboos and trees, we searched and collected data from articles published before February 13, 2020 by using Google Academic (Google Inc., Mountain View, CA, USA) and Baidu Academic (Baidu Inc., Beijing, China). The searching keywords included ‘stem CO_2_ flux’, ‘stem respiration’, ‘woody tissue respiration’, ‘wood CO_2_ efflux’, ‘woody tissue efflux’, ‘Cuticular photosynthesis’, and ‘woody tissue photosynthesis’. From the collected articles, a secondary selection was conducted to extract the following data: 1) *E*_s_ produced by infrared gas analyzer; 2) mean seasonal *E*_s_; 3) air temperature measured at the same time with the *E*_s_ measurement. Finally, we got the following variables from the selected articles: time range for the *E*_s_ measurement, mean *E*_s_, mean air temperature, tree species, latitude, and longitude of the study site. latitudes and longitudes of some studies was obtained from the Baidu Map (<http://api.map.baidu.com/lbsapi/getpoint/index.html>) according to the sites provided in these studies.

According to the latitude, the dataset was classified into three categories: temperate (latitude > 40^o^), subtropical (23.5^o^ < latitude < 40^o^) and tropical (0^o^ < latitude <23.5^o^) forests. Data from controlled experiments (e.g., increasing CO_2_concentration, heating soil or air, pruning, and annulus stripping) were treated with the following methods: 1) an intergroup mean *E*_s_ was applied if there was no significant difference between control and treated groups; 2) the control group mean *E*_s_ was used if there was a significant difference between control and treated groups. The units of *E*_s_ in some literature were not consistent with other literature, so it was necessary to retrieve data from literature and convert *E*_s_ to a unified unit, i.e., mol m^-2^ s^-1^, according to the size and installation of the chambers.

To identify whether the selected tree species have stem photosynthesis or not, articles about cuticular photosynthesis were searched with the following keywords: ‘Culm photosynthesis’, ‘Cuticular photosynthesis’ and ‘woody tissue photosynthesis’. Families, tree species, and cuticular photosynthesis rates were retrieved from the selected articles (Table S2), and they were furtherly matched and merged with the synthesized *E*_s_ data (Table S1) by family names. In total, there were 195 tree species collected in Table S1, among which most of the species is distributed across North America, Europe, and East Asia.

Table S1 The stem respiration data of different species

| Phylum | 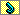Family | Species | Location | Temperature Zone | Observation Time (Year, Month) | Cuticular Photosynthesis | Temperature (℃) | CO_2_ efflux （μmol m^-2^ s^-1^） | Reference |
| --- | --- | --- | --- | --- | --- | --- | --- | --- | --- |
| Gymnospermae | Cupressaceae | *Chamaecyparis obtusa* | 35°12'N | Subtropical | 1991 - 1996, 6 | Unknown | 15.05 | 7.8 | (Adu-Bredu) |
|  |  |  | 136°59'E |  |  |  |  |  |  |
| Gymnospermae | Cupressaceae | *Chamaecyparis obtusa* | 36°14'N  140°11'E | Subtropical | 2001 - 2003 | Unknown | 13.20 | 5.87 | (Araki *et al.*, 2015) |
| Gymnospermae | Cupressaceae | *Chamaecyparis obtusa var. formosana* | 24°35'N  121°25'E | Subtropical | 2009, 5 | Unknown | 14.23 | 1.03 | (Yang *et al.*, 2014) |
| Gymnospermae | Pinaceae | *Pseudotsuga menziesii* | 47°11'N  116°17'W | Temperate | 2008, 6 - 7 | Unknown | 14.16 | 7.43 | (Ubierna et al., 2009) |
| Gymnospermae | Pinaceae | *Larix gmelinii* | 45°20'N  127°34'E | Temperate | 2005 - 2006 | Yes | 14.79 | 7.34 | (Wang *et al.*, 2014) |
| Gymnospermae | Pinaceae | *Abies grandis* | 47°11'N  116°17'W | Temperate | 2007, 9 | Unknown | 20.61 | 5.06 | (Ubierna *et al.*, 2009) |
| Gymnospermae | Pinaceae | *Pinus koraiensis* | 42°24'N  128°6'E | Temperate | 2003, 5 - 9 | Yes | 16.62 | 4.14 | (Miao *et al.*, 2005) |
| Gymnospermae | Pinaceae | *Larix gmelinii* | 45°20'N  127°34'E | Temperate | 2007, 6 | Yes | 19.30 | 3.78 | (Wang *et al.*, 2011) |
| Gymnospermae | Pinaceae | *Pinus koraiensis* | 42°24'N  128°6'E | Temperate | 2003, 5 - 9 | Yes | 16.62 | 3.76 | (Miao *et al.*, 2005) |
| Gymnospermae | Pinaceae | *Pinus canariensis* | 28°18'N  16°34'W | Subtropical | 2008, 12 | Yes | 11.30 | 3.74 | (Brito *et al.*, 2010) |
| Gymnospermae | Pinaceae | *Pinus massoniana* | 26°44'N  115°4'E | Subtropical | 2015, 3 - 11 | Yes | 24.70 | 3.26 | (Jie *et al.*, 2017) |
| Gymnospermae | Pinaceae | *Pinus cembra* | 47°13'N  11°27'E | Temperate | 2006 - 2007, 6 - 8 | Yes | 9.65 | 2.62 | (Gruber *et al.*, 2009) |
| Gymnospermae | Pinaceae | *Larix gmelinii* | 45°20'N  127°34'E | Temperate | 2001 - 2002, 08 - 07 | Yes | 19.54 | 2.41 | (Wang *et al.*, 2003) |
| Gymnospermae | Pinaceae | *Pinus strobus* | 42°42'N  80°22'W | Temperate | 2006, 1-12 | Yes | 23.97 | 2.37 | (Khomik *et al.*, 2010) |
| Gymnospermae | Pinaceae | *Picea koraiensis* | 45°24'N  127°40'E | Temperate | 2009, 7 - 10 | Yes | 22.00 | 2.21 | (Xu *et al.*, 2011) |
| Gymnospermae | Pinaceae | *Pinus ponderosa* | 44°30'N  121°37'W | Temperate |  | Yes | 17.00 | 2.20 | (Law *et al.*, 1999) |
| Gymnospermae | Pinaceae | *Larix principis-rupprechtii* | 42°24'N  117°15'E | Temperate | 2010 - 2012 | Yes | 14.44 | 2.13 | (Yang *et al.*, 2014) |
| Gymnospermae | Pinaceae | *Pinus cembra* | 47°N  11°E | Temperate | 2001 - 2003, 1 - 10 | Yes | 8.39 | 1.94 | (Wieser & Bahn, 2004) |
| Gymnospermae | Pinaceae | *Pinus sylvestris var． mongolica* | 45°24'N  127°40'E | Temperate | 2009, 1 - 6 | Yes | 22.00 | 1.52 | (Xu *et al.*, 2011) |
| Gymnospermae | Pinaceae | *Pinus densiflora* | 34°23'N  132°39'E | Subtropical | 2003, 9 | Yes | 22.18 | 1.52 | (Kim & Nakane, 2005) |
| Gymnospermae | Pinaceae | *Pinus koraiensis* | 42°24'N  128°6'E | Temperate | 2003, 5 | Yes | 16.62 | 1.47 | (Wang *et al.*, 2005) |
| Gymnospermae | Pinaceae | *Pinus massoniana* | 31°46'N  114°2'E | Subtropical | 2009 - 2010, 01, 04, 07, 10 | Yes | 17.59 | 1.28 | (Chi *et al.*, 2020a) |
| Gymnospermae | Pinaceae | *Pinus taeda* | 31°46'N  114°2'E | Subtropical | 2009 - 2010, 01, 04,07,10 | Yes | 17.17 | 1.27 | (Chi *et al.*, 2020a) |
| Gymnospermae | Pinaceae | *Pinus sylvestris* | 62°52'N  30°49'E | Temperate | 2001, 2002, 2003, 4 - 9 | Yes | 9.92 | 1.09 | (Zha, 2004) |
| Gymnospermae | Pinaceae | *Pinus caribaea* | 29°44'N  82°9'W | Subtropical |  | yes | 14.50 | 0.83 | (Asao *et al.*, 2015) |
| Gymnospermae | Pinaceae | *Pinus ponderosa* | 46°51'N  113°29'W | Temperate |  | yes | 7.50 | 0.80 | (Ryan *et al.*, 1995b) |
| Gymnospermae | Pinaceae | *Larix gmelinii* | 45°20'N  127°34'E | Temperate | 2006, 9 | yes | 16.50 | 0.74 | (Wang *et al.*, 2011) |
| Gymnospermae | Pinaceae | *Pinus koraiensis* | 46°10'N  89°40'W | Temperate |  | yes | 10.50 | 0.71 | (Ryan *et al.*, 1995b) |
| Gymnospermae | Pinaceae | *Pinus canariensis* | 28°18'N  16°34'W | Subtropical | 2008, 6 | yes | 23.80 | 0.51 | (Brito *et al.*, 2010) |
| Gymnospermae | Pinaceae | *Tsuga heterophylla* | 45°45'N  122°35'W | Temperate |  | Unknown | 8.50 | 0.48 | (Ryan *et al.*, 1995a) |
| Gymnospermae | Pinaceae | *Pinus massoniana* | 26°44'N  115°4'E | Subtropical | 2015, 01 - 02 , 12 | yes | 11.10 | 0.39 | (Jie et al., 2017) |
| Gymnospermae | Pinaceae | *Picea mariana* | 55°53'N  98°20'W | Temperate | 2005 - 2007, 6 - 9 | yes | 2.89 | 0.32 | (Bronson & Gower, 2010) |
| Gymnospermae | Pinaceae | *Abies fabri* | 29°34'N  102°59'E | Subtropical | 2014, 11 - 12 | Unknown | 4.00 | 0.18 | (Zhao *et al.*, 2018) |
| Gymnospermae | Taxodiaceae | *Cunninghamia lanceolata* | 27°N  110°E | Subtropical | 2012 - 2013, 9 - 12 | Unknown | 16.00 | 1.67 | (Yang *et al.*, 2015) |
| Angiospermae | Hamamelidaceae | *Liquidambar styraciflua* | 33°N  84°W | Subtropical | 2002, 10 - 11 | Unknown | 12.52 | 5.70 | (A & Teskey, 2004) |
| Angiospermae | Moraceae | *Ficus altissima* | 26°2'N  119°18'E | Subtropical | 2008, 5 - 6 | Unknown | 25.50 | 14.01 | (Wei *et al.*, 2009) |
| Angiospermae | Moraceae | *Ficus altissima* | 26°2'N  119°18'E | Subtropical | 2007, 11 | Unknown | 19.10 | 6.86 | (Wei *et al.*, 2009) |
| Angiospermae | Moraceae | *Ficus sp* | 4°7'S  78°58'W | Tropics | 2005, 10 - 12 | Unknown | 20.80 | 1.74 | (Zach *et al.*, 2008) |
| Angiospermae | Oleaceae | *Fraxinus chinensis* | 45°43'N  126°38'E | Temperate | 2008, 9 | Yes | 20.84 | 8.39 | (Wang *et al.*, 2019) |
| Angiospermae | Oleaceae | *Fraxinus mandshurica* | 45°20'N  127°34'E | Temperate | 2007, 6 | Yes | 18.60 | 4.98 | (Wang *et al.*, 2011) |
| Angiospermae | Oleaceae | *Fraxinus pennsylvanica* | 45°48'N  90°7'W | Temperate | 2002, 5 - 11 | Yes | 16.77 | 3.49 | (Bolstad *et al.*, 2004) |
| Angiospermae | Oleaceae | *Fraxinus mandshurica* | 45°20'N  127°34'E | Temperate | 2006, 9 | Yes | 15.80 | 1.98 | (Wang *et al.*, 2011) |
| Angiospermae | Platanaceae | *Platanus occidentalis* | 33°N  84°W | Subtropical | 2002, 10 - 11 | Unknown | 11.13 | 2.60 | (A & Teskey, 2004) |
| Angiospermae | Poaceae | *Phyllostachys* | 8°31'N  76°54'E | Tropics | 2014, 3 | Yes | 29.29 | 9.12 | (Zachariah *et al.*, 2016) |
| Angiospermae | Poaceae | *Phyllostachys* | 30°15'N  119°42'E | Subtropical | 2017, 5 - 6 | Yes | 24.39 | 6.32 |  |
| Angiospermae | Poaceae | *Phyllostachys* | 30°15'N  119°42'E | Subtropical | 2017, 7 - 9 | Yes | 28.06 | 1.83 |  |
| Angiospermae | Poaceae | *Phyllostachy edulis* | 27°30'N  114°20'E | Subtropical | 2008 - 2009, 7 - 9 | Yes | 17.40 | 1.58 | (Xiao *et al.*, 2010) |
| Angiospermae | Betulaceae | *Betula platyphylla* | 45°43'N  126°38'E | Temperate | 2008, 9 | Yes | 17.63 | 6.70 | (Wang *et al.*, 2019) |
| Angiospermae | Betulaceae | *Betula platyphylla* | 45°20'N  127°34'E | Temperate | 2007, 6 | Yes | 20.00 | 3.32 | (Wang *et al.*, 2011) |
| Angiospermae | Betulaceae | *Betula costata* | 45°24'N  127°40'E | Temperate | 2009, 6 - 10 | Yes | 22.00 | 1.63 | (Xu *et al.*, 2011) |
| Angiospermae | Betulaceae | *Betula platyphylla* | 45°20'N  127°34'E | Temperate | 2006, 9 | Yes | 15.20 | 0.82 | (Wang *et al.*, 2011) |
| Angiospermae | Fagaceae | *Quercus prinus* | 35°58'N  84°17'W | Subtropical | 1993, 3 - 10 | Unknown | 12.32 | 3.78 | (A and Teskey, 2004) |
| Angiospermae | Fagaceae | *Quercus alba* | 35°58'N  84°17'W | Subtropical | 1993, 3 - 10 | Yes | 11.21 | 3.59 | (Edwards & Hanson, 1996) |
| Angiospermae | Fagaceae | *Fagus grandifolia* | 33°54'N  77°24'W | Subtropical | 2002, 10 - 11 | Yes | 14.20 | 3.50 | (A & Teskey, 2004) |
| Angiospermae | Fagaceae | *Quercus mongolica* | 45°24'N  127°40'E | Temperate | 2009, 6 - 10 | Yes | 22.00 | 2.45 | (Xu *et al.*, 2011) |
| Angiospermae | Fagaceae | *Quercus ilex* | 43°44'N  3°35'E | Temperate | 2011, 3 - 11 | Yes | 16.25 | 1.61 | (Rodríguez-Calcerrada *et al.*, 2014) |
| Angiospermae | Fagaceae | *Quercus palustris* | 31°29'N  114°2'E | Subtropical | 2009 - 2010, 01, 04, 07, 10 | Yes | 16.8 | 1.24 | (Chi *et al.*, 2020a) |
| Angiospermae | Fagaceae | *Quercus serrat* | 34°47'N  135°50'E | Subtropical |  | Yes | 15.20 | 0.84 | (Miyama *et al.*, 2006) |
| Angiospermae | Fagaceae | *Castanopsis rufescens* | 24°32'N  102°2'E | Subtropical | 2009, 5 - 10 | Unknown | 15.17 | 0.75 | (Hu & Sha, 2010) |
| Angiospermae | Fagaceae | *Castanopsis rufescens* | 24°32'N  102°2'E | Subtropical | 2009, 11 - 4 | Unknown | 10.34 | 0.58 | (Hu & Sha, 2010) |
| Angiospermae | Lauraceae | *Cinnamomum camphora (Linn) Presl* | 26°2'N  119°18'E | Subtropical | 2008, 5 - 6 | Unknown | 25.89 | 10.59 | (Wang *et al.*, 2011) |
| Angiospermae | Lauraceae | *Cinnamomum camphora (Linn) Presl* | 26°2'N  119°18'E | Subtropical | 2007, 11 | Unknown | 19.10 | 4.35 | (Wang *et al.*, 2011) |
| Angiospermae | Lauraceae | *Nectandra sp* | 4°7'S  78°58'W | Tropics | 2005, 10-12 | Unknown | 17.20 | 2.16 | (Zach *et al.*, 2008) |
| Angiospermae | Lauraceae | *Endlicheria oreocola* | 4°7'S  78°58'W | Tropics | 2005, 10 - 12 | Unknown | 17.20 | 0.83 | (Zach *et al.*, 2008) |
| Angiospermae | Lauraceae | *Machilus bombycina* | 24°32'N  102°2'E | Subtropical | 2009, 5 - 10 | unknown | 15.50 | 0.51 | (Hu & Sha, 2010) |
| Angiospermae | Lauraceae | *Machilus bombycina* | 24°32'N  102°2'E | Subtropical | 2009, 11 - 4 | unknown | 13.5 | 0.24 | (Hu & Sha, 2010) |
| Angiospermae | Salicaceae | *P. nigra* | 42°22'N  11°48'E | Temperate | 2001, 5 | Yes | 17.72 | 4.64 | (Gielen *et al.*, 2003) |
| Angiospermae | Salicaceae | *P. alba* | 42°22'N  11°48'E | Temperate | 2001, 5 | Yes | 18.04 | 4.36 | (Gielen *et al.*, 2003) |
| Angiospermae | Salicaceae | *Populus × canadensis* | 43°44'N  3°35'E | Temperate | 2014, 4 | Yes | 24.50 | 3.92 | (Salomón *et al.*, 2016) |
| Angiospermae | Salicaceae | *Populus deltoides* | 33°N  84°W | Subtropical | 2006, 10 - 11 | Yes | 16.07 | 3.44 | (Saveyn et al., 2008) |
| Angiospermae | Salicaceae | *P. ×euramericana* | 42°22'N  11°48'E | Temperate | 2001, 5 | Yes | 18.43 | 3.34 | (Gielen *et al.*, 2003) |
| Angiospermae | Salicaceae | *Populus × canadensis* | 43°44'N  3°35'E | Temperate | 2014, 4 | Yes | 25.50 | 3.26 | (Salomón *et al.*, 2020) |
| Angiospermae | Salicaceae | *Populus tremuloides* | 45°33'N  84°40'W | Temperate | 1995 - 1996, 6 - 7 | Yes | 28.00 | 2.59 | (Marler, 2020) |
| Angiospermae | Salicaceae | *Populus tremuloides* | 45°29'N  90°4'W | Temperate | 2002, 5 - 11 | Yes | 16.77 | 2.23 | (Bolstad *et al.*, 2004) |
| Angiospermae | Salicaceae | *Populus davidiana* | 45°24'N  127°40'E | Temperate | 2009, 6 - 10 | Yes | 22.00 | 1.96 | (Xu *et al.*, 2011) |
| Angiospermae | Salicaceae | *Populus × canadensis* | 43°44'N  3°35'E | Temperate | 2014, 4 | Yes | 22.20 | 1.73 | (Salomón *et al.*, 2020) |
| Angiospermae | Salicaceae | *Populus tremuloides* | 53°37'N  106°12'W | Temperate | 2001 - 2003 | Yes | -5.60 | 0.79 | (Gaumont-Guay *et al.*, 2006) |
| Angiospermae | Anacardiaceae | *Mangifera indica* | 26°2'N  119°18'E | Subtropical | 2007, 11 | Unknown | 19.10 | 5.14 | (Wei *et al.*, 2009) |
| Angiospermae | Anacardiaceae | *Mangifera indica* | 26°2'N  119°18'E | Subtropical | 2008, 5 - 6 | Unknown | 25.50 | 4.14 | (Wei *et al.*, 2009) |
| Angiospermae | Anacardiaceae | *Tapirira guianensis* | 5°17'N  52°54'W | Tropics | 2008, 3 - 7 | Unknown | 24.50 | 1.40 | (Stahl *et al.*, 2011a) |
| Angiospermae | Anacardiaceae | *Tapirira guianensis* | 5°17'N  52°54'W | Tropics | 2008, 9 - 12 | Unknown | 25.60 | 0.70 | (Stahl *et al.*, 2011a) |
| Angiospermae | Sapindaceae | *Acer saccharum* | 45°29'N  90°4'W | Temperate | 2002, 5 - 11 | Yes | 16.77 | 2.49 | (Bolstad *et al.*, 2004) |
| Angiospermae | Sapindaceae | *Acer mono* | 45°24'N  127°40'E | Temperate | 2009, 6 - 10 | Yes | 22.00 | 2.17 | (Xu *et al.*, 2011) |
| Angiospermae | Sapindaceae | *Acer rubrum* | 35°58'N  84°17'W | Subtropical | 1993, 3 - 10 | Yes | 12.34 | 1.79 | (Zach et al., 2008) |
| Angiospermae | Sapindaceae | *Matayba inelegans* | 4°7'S  78°58'W | Tropics | 2005, 10 - 12 | Unknown | 17.20 | 0.97 | (Zach *et al.*, 2008) |
| Angiospermae | Theaceae | *Schima superba* | 23°8'N  113°E | Tropics | 2009, 7 | Unknown | 30.34 | 6.53 | (Yang *et al.*, 2015) |
| Angiospermae | Theaceae | *Schima superba* | 23°8'N  113°E | Tropics | 2009, 10 - 11 | Unknown | 25.02 | 3.81 | (Yang *et al.*, 2015) |
| Angiospermae | Theaceae | *Schima noronhae* | 24°32'N  102°2'E | Subtropical | 2009, 5 - 10 | Unknown | 15.40 | 0.46 | (Hu & Sha, 2010) |
| Angiospermae | Theaceae | *Schima noronhae* | 24°32'N  102°2'E | Subtropical | 2009, 11 - 4 | Unknown | 11.50 | 0.24 | (Hu & Sha, 2010) |
| Angiospermae | Juglandaceae | *Juglans mandshurica* | 45°24'N  127°40'E | Temperate | 2009, 6 - 10 | Yes | 22.00 | 2.30 | (Xu *et al.*, 2011) |
| Angiospermae | Fabaceae | *Bauhinia* | 26°2'N  119°18'E | Subtropical | 2007, 11 | Unknown | 19.10 | 10.52 | (Wei *et al.*, 2009) |
| Angiospermae | Fabaceae | *Tachigali paniculata* | 3°50'N  73°22'W | Tropics | 2010, 9 | Unknown | 26.70 | 5.20 | (Angert *et al.*, 2012) |
| Angiospermae | Fabaceae | *Bauhinia* | 26°2'N  119°18'E | Subtropical | 2008, 5 - 6 | Unknown | 25.50 | 4.27 | (Wei *et al.*, 2009) |
| Angiospermae | Fabaceae | *Hymenolobium pulcherrimum* | 3°50'N  73°22'W | Tropics | 2010, 9 | Unknown | 26.70 | 4.17 | (Angert *et al.*, 2012) |
| Angiospermae | Fabaceae | *Hymenolobium pulcherrimum* | 3°50'N  73°22'W | Tropics | 2011, 4 | Unknown | 26.70 | 3.60 | (Angert *et al.*, 2012) |
| Angiospermae | Fabaceae | *Tachigali paniculata* | 3°50'N  73°22'W | Tropics | 2011, 4 | Unknown | 26.70 | 3.00 | (Angert *et al.*, 2012) |
| Angiospermae | Fabaceae | *Dicorynia guianensis* | 5°17'N  52°54'W | Tropics | 2008, 3 - 7 | Unknown | 24.50 | 2.08 | (Stahl *et al.*, 2011a) |
| Angiospermae | Fabaceae | *Recordoxylon speciosum* | 5°17'N  52°54'W | Tropics | 2008, 3 - 7 | Unknown | 24.50 | 1.60 | (Stahl *et al.*, 2011a) |
| Angiospermae | Fabaceae | *Recordoxylon speciosum* | 5°17'N  52°54'W | Tropics | 2008, 3 - 7 | Unknown | 24.50 | 1.59 | (Stahl *et al.*, 2011a) |
| Angiospermae | Fabaceae | *Pentaclethra macroloba* | 10°26'N  84°W | Tropics | 2009 - 2010 | Unknown | 25.00 | 1.40 | (Asao *et al.*, 2015) |
| Angiospermae | Fabaceae | *Vouacapoua americana* | 5°17'N  52°54'W | Tropics | 2008, 3 - 7 | Unknown | 24.50 | 1.39 | (Stahl et al., 2011) |
| Angiospermae | Fabaceae | *Eperua falcata* | 5°17'N  52°54'W | Tropics | 2008, 3 - 7 | Unknown | 24.50 | 1.20 | (Stahl *et al.*, 2011a) |
| Angiospermae | Fabaceae | *Recordoxylon speciosum* | 5°17'N  52°54'W | Tropics | 2008, 9 - 12 | Unknown | 25.60 | 1.17 | (Stahl *et al.*, 2011a) |
| Angiospermae | Fabaceae | *Swartzia polyphylla* | 5°17'N  52°54'W | Tropics | 2008, 3 - 7 | Unknown | 24.50 | 1.15 | (Stahl *et al.*, 2011a) |
| Angiospermae | Fabaceae | *Recordoxylon speciosum* | 5°17'N  52°54'W | Tropics | 2008, 9 - 12 | Unknown | 25.60 | 1.11 | (Stahl *et al.*, 2011a) |
| Angiospermae | Fabaceae | *Dicorynia guianensis* | 5°17'N  52°54'W | Tropics | 2008, 9 - 12 | Unknown | 25.6 | 1.03 | (Stahl *et al.*, 2011a) |
| Angiospermae | Fabaceae | *Eperua falcata* | 5°17'N  52°54'W | Tropics | 2008, 9 - 12 | Unknown | 25.60 | 0.83 | (Stahl *et al.*, 2011a) |
| Angiospermae | Fabaceae | *Vouacapoua americana* | 5°17'N  52°54'W | Tropics | 2008, 9 - 12 | Unknown | 25.60 | 0.80 | (Stahl *et al.*, 2011a) |
| Angiospermae | Fabaceae | *Pentaclethra. macroloba* | 10°20'N  83°50'W | Tropics | 2003 - 2005 | Unknown | 25.00 | 0.63 | (Stahl et al., 2011) |
| Angiospermae | Fabaceae | *Swartzia polyphylla* | 5°17'N  52°54'W | Tropics | 2008, 9 - 12 | Unknown | 25.60 | 0.41 | (Stahl *et al.*, 2011a) |
| Angiospermae | Ulmaceae | *Ulmus propinqua* | 45°24'N  127°40'E | Tempera | 2009, 6 - 10 | Yes | 22.00 | 1.99 | (Xu *et al.*, 2011) |
| Angiospermae | Simaroubaceae | *Simarouba amara* | 3°50'N  73°22'W | Tropics | 2010, 9 | Unknown | 26.70 | 2.80 | (Stahl et al., 2011) |
| Angiospermae | Simaroubaceae | *Simarouba amara* | 3°50'N  73°22'W | Tropics | 2011, 4 | Unknown | 26.70 | 2.00 | (Stahl et al., 2011) |
| Angiospermae | Apocynaceae | *Aspidosperma album* | 5°17'N  52°54'W | Tropics | 2008, 9 - 12 | Unknown | 25.60 | 2.31 | (Stahl *et al.*, 2011a) |
| Angiospermae | Apocynaceae | *Aspidosperma album* | 5°17'N  52°54'W | Tropics | 2008, 3 - 7 | Unknown | 24.50 | 1.91 | (Stahl *et al.*, 2011a) |
| Angiospermae | Humiriaceae | *Vantanea sp.* | 5°17'N  52°54'W | Tropics | 2008, 9 - 12 | Unknown | 25.60 | 2.12 | (Stahl *et al.*, 2011a) |
| Angiospermae | Humiriaceae | *Vantanea sp.* | 5°17'N  52°54'W | Tropics | 2008, 3 - 7 | Unknown | 24.50 | 1.57 | (Stahl *et al.*, 2011a) |
| Angiospermae | Dipterocarpaceae | *Shorea beccariana* | 4°12'N  114°2'E | Tropics | 2012, 1 - 9 | Unknown | 28.03 | 2.02 | (Ayumi *et al.*, 2014) |
| Angiospermae | Dipterocarpaceae | *Dryobalanops aromatica* | 4°12'N  114°2'E | Tropics | 2012, 1 - 9 | Unknown | 28.03 | 1.77 | (Ayumi *et al.*, 2014) |
| Angiospermae | Chrysobalanaceae | *Hirtella bicornis* | 5°17'N  52°54'W | Tropics | 2008, 9 - 12 | Unknown | 25.60 | 2.94 | (Stahl *et al.*, 2011a) |
| Angiospermae | Chrysobalanaceae | *Hirtella bicornis* | 5°17'N  52°54'W | Tropics | 2008, 3 - 7 | Unknown | 24.50 | 2.88 | (Stahl *et al.*, 2011a) |
| Angiospermae | Chrysobalanaceae | *Licania membranacea* | 5°17'N  52°54'W | Tropics | 2008, 9 - 12 | Unknown | 25.60 | 1.45 | (Stahl *et al.*, 2011a) |
| Angiospermae | Chrysobalanaceae | *Licania alba* | 5°17'N  52°54'W | Tropics | 2008, 3 - 7 | Unknown | 24.50 | 1.40 | (Stahl *et al.*, 2011a) |
| Angiospermae | Chrysobalanaceae | *Licania membranacea* | 5°17'N  52°54'W | Tropics | 2008, 3 - 7 | Unknown | 24.50 | 1.25 | (Stahl *et al.*, 2011a) |
| Angiospermae | Chrysobalanaceae | *Licania heteromorpha* | 5°17'N  52°54'W | Tropics | 2008, 9 - 12 | Unknown | 25.60 | 1.24 | (Stahl *et al.*, 2011a) |
| Angiospermae | Chrysobalanaceae | *Licania heteromorpha* | 5°17'N  52°54'W | Tropics | 2008, 3 - 7 | Unknown | 24.50 | 1.22 | (Stahl *et al.*, 2011a) |
| Angiospermae | Chrysobalanaceae | *Licania alba* | 5°17'N  52°54'W | Tropics | 2008, 9 - 12 | Unknown | 25.60 | 1.06 | (Stahl *et al.*, 2011a) |
| Angiospermae | Sterculiaceae | *Sterculia pruriens* | 5°17'N  52°54'W | Tropics | 2008, 3 - 7 | Unknown | 24.50 | 2.02 | (Stahl *et al.*, 2011a) |
| Angiospermae | Sterculiaceae | *Sterculia pruriens* | 5°17'N  52°54'W | Tropics | 2008, 9 - 12 | Unknown | 25.6 | 1.24 | (Stahl *et al.*, 2011a) |
| Angiospermae | Lecythidaceae | *Lecythis zabucajo* | 5°17'N  52°54'W | Tropics | 2008, 3 - 7 | Unknown | 24.50 | 4.04 | (Stahl *et al.*, 2011a) |
| Angiospermae | Lecythidaceae | *Bertholletia excelsa* | 1°43'S  51°27'W | Tropics | 2009 - 2010, 7 - 12 | Unknown | 26.67 | 2.10 | (Doughty *et al.*, 2014) |
| Angiospermae | Lecythidaceae | *Lecythis zabucajo* | 5°17'N  52°54'W | Tropics | 2008, 9 - 12 | Unknown | 25.60 | 1.64 | (Stahl *et al.*, 2011a) |
| Angiospermae | Lecythidaceae | *Eschweilera sagotiana* | 5°17'N  52°54'W | Tropics | 2008, 3 - 7 | Unknown | 24.50 | 1.26 | (Stahl *et al.*, 2011a) |
| Angiospermae | Lecythidaceae | *Lecythis poiteaui* | 5°17'N  52°54'W | Tropics | 2008, 3 - 7 | Unknown | 24.50 | 1.03 | (Stahl *et al.*, 2011a) |
| Angiospermae | Lecythidaceae | *Lecythis poiteaui* | 5°17'N  52°54'W | Tropics | 2008, 9 - 12 | Unknown | 25.60 | 0.70 | (Stahl *et al.*, 2011a) |
| Angiospermae | Lecythidaceae | *Eschweilera sagotiana* | 5°17'N  52°54'W | Tropics | 2008, 9 - 12 | Unknown | 25.60 | 0.63 | (Stahl *et al.*, 2011a) |
| Angiospermae | Melastomataceae | *Miconia punctata* | 4°7'S  78°58'W | Tropics | 2005, 10 - 12 | Unknown | 20.80 | 2.01 | (Zach *et al.*, 2008) |
| Angiospermae | Melastomataceae | *Miconia punctata* | 4°7'S  78°58'W | Tropics | 2005, 10 - 12 | Unknown | 17.20 | 0.95 | (Zach *et al.*, 2008) |
| Angiospermae | Melastomataceae | *Graffenrieda emarginata* | 4°7'S  78°58'W | Tropics | 2005, 10 - 12 | Unknown | 17.20 | 0.71 | (Zach *et al.*, 2008) |
| Angiospermae | Myrtaceae | *Eucalyptus globulus* | 38°22'N  8°19'W | Subtropical | 2007, 10 | Yes | 20.30 | 3.40 | (Cerasoli *et al.*, 2009) |
| Angiospermae | Myrtaceae | *Eucalyptus tetrodonta* | 12°21'S  131°5'E | Tropics | 2001, 11 - 4 | Unknown | 26.10 | 0.54 | (Chen *et al.*) |
| Angiospermae | Myrtaceae | *Eucalyptus tetrodonta* | 12°21'S  131°5'E | Tropics | 2001, 5 - 10 | Unknown | 23.4 | 0.1 | (Chen *et al.*) |
| Angiospermae | Malvaceae | *Tilia amurensis* | 45°24'N  127°40'E | Temperate | 2009, 6 - 10 | Yes | 22.00 | 1.58 | [89] |
| Angiospermae | Malvaceae | *Sterculia pruriens* | 5°17'N  52°54'W | Tropics | 2008, 9 - 12 | Unknown | 25.60 | 1.26 | (Stahl *et al.*, 2011a) |
| Angiospermae | Malvaceae | *Sterculia pruriens* | 5°17'N  52°54'W | Tropics | 2008, 3 - 7 | Unknown | 24.50 | 1.24 | (Stahl *et al.*, 2011a) |
| Angiospermae | Malvaceae | *Tilia　americana* | 45°29'N  90°4'W | Temperate | 2002, 5 - 11 | Yes | 16.77 | 1.23 | (Bolstad *et al.*, 2004) |
| Angiospermae | Phyllanthaceae | *Hieronyma alchorneoides* | 10°26'N  84°W | Tropics | 2009 - 2010 | Unknown | 25.00 | 1.52 | (Asao *et al.*, 2015) |
| Angiospermae | Sapotaceae | *Chrysophyllum sanguinolentum* | 5°17'N  52°54'W | Tropics | 2008, 3 - 7 | Unknown | 24.50 | 2.17 | (Stahl *et al.*, 2011a) |
| Angiospermae | Sapotaceae | *Pouteria cf* | 4°7'S  78°58'W | Tropics | 2005, 10 - 12 | Unknown | 20.80 | 1.78 | (Zach *et al.*, 2008) |
| Angiospermae | Sapotaceae | *Pradosia cochlearia* | 5°17'N  52°54'W | Tropics | 2008, 3 - 7 | Unknown | 24.50 | 1.46 | (Stahl *et al.*, 2011a) |
| Angiospermae | Sapotaceae | *Chrysophyllum sanguinolentum* | 5°17'N  52°54'W | Tropics | 2008, 9 - 12 | Unknown | 25.60 | 1.33 | (Stahl *et al.*, 2011a) |
| Angiospermae | Sapotaceae | *Micropholis guyanensis* | 4°7'S  78°58'W | Tropics | 2005, 10 - 12 | Unknown | 17.20 | 1.03 | (Zach *et al.*, 2008) |
| Angiospermae | Sapotaceae | *Pradosia cochlearia* | 5°17'N  52°54'W | Tropics | 2008, 9 - 12 | Unknown | 25.60 | 0.97 | (Stahl *et al.*, 2011a) |
| Angiospermae | Sapotaceae | *Chrysophyllum sp* | 4°7'S  78°58'W | Tropics | 2005, 10 - 12 | Unknown | 20.80 | 0.87 | (Zach *et al.*, 2008) |
| Angiospermae | Rutaceae | *Phellodendron amurense* | 45°24'N  127°40'E | Temperate | 2009, 6 - 10 | Yes | 22.00 | 1.32 | (Xu *et al.*, 2011) |
| Angiospermae | Arecaceae | *Prestoea montana* | 18°N  65°W | Tropics | 2005, 3 | Unknown | 22.15 | 1.27 | (Chi *et al.*, 2020b) |
| Angiospermae | Clethraceae | *Clethra revoluta cf* | 3°58'S  79°4'W | Tropics | 2005, 10-12 | Unknown | 17.20 | 0.94 | (Zach *et al.*, 2008) |
| Angiospermae | Goupiaceae | *Goupia glabra* | 5°17'N  52°54'W | Tropics | 2008, 3 - 7 | Unknown | 24.50 | 1.70 | (Stahl *et al.*, 2011a) |
| Angiospermae | Goupiaceae | *Goupia glabra* | 5°17'N  52°54'W | Tropics | 2008, 9 - 12 | Unknown | 25.60 | 1.45 | (Stahl *et al.*, 2011a) |
| Angiospermae | Goupiaceae | *Goupia glabra* | 5°17'N  52°54'W | Tropics | 2008, 3 - 7 | Unknown | 24.50 | 1.43 | (Stahl *et al.*, 2011a) |
| Angiospermae | Goupiaceae | *Goupia glabra* | 5°17'N  52°54'W | Tropics | 2008, 9 - 12 | Unknown | 25.60 | 0.83 | (Stahl *et al.*, 2011a) |
| Angiospermae | Clusiaceae | *Moronobea coccinea* | 5°17'N  52°54'W | Tropics | 2008, 3 - 7 | Unknown | 24.50 | 2.01 | (Stahl *et al.*, 2011a) |
| *Angiospermae* | *Clusiaceae* | *Moronobea coccinea* | 5°17'N  52°54'W | Tropics | 2008, 3 - 7 | Unknown | 24.50 | 1.71 | (Stahl *et al.*, 2011a) |
| *Angiospermae* | *Clusiaceae* | *Symphonia sp.* | 5°17'N  52°54'W | Tropics | 2008, 3 - 7 | Unknown | 24.50 | 1.50 | (Stahl *et al.*, 2011a) |
| *Angiospermae* | *Clusiaceae* | *Symphonia sp.* | 5°17'N  52°54'W | Tropics | 2008, 9 - 12 | Unknown | 25.60 | 1.13 | (Stahl *et al.*, 2011a) |
| *Angiospermae* | *Clusiaceae* | *Moronobea coccinea* | 5°17'N  52°54'W | Tropics | 2008, 9 - 12 | Unknown | 25.60 | 1.09 | (Stahl *et al.*, 2011a) |
| *Angiospermae* | *Clusiaceae* | *Moronobea coccinea* | 5°17'N  52°54'W | Tropics | 2008, 9 - 12 | Unknown | 25.60 | 0.77 | (Stahl *et al.*, 2011a) |
| *Angiospermae* | *Clusiaceae* | *Clusia sp* | 4°6'S  79°10'W | Tropics | 2005, 10 - 12 | Unknown | 10.60 | 0.15 | (Zach *et al.*, 2008) |
| *Angiospermae* | *Myristicaceae* | *Virola michelii* | 5°17'N  52°54'W | Tropics | 2008, 3 - 7 | Unknown | 24.50 | 1.49 | (Stahl *et al.*, 2011a) |
| *Angiospermae* | *Myristicaceae* | *Virola surinamensis* | 5°17'N  52°54'W | Tropics | 2008, 9 - 12 | Unknown | 25.60 | 1.41 | (Stahl *et al.*, 2011a) |
| *Angiospermae* | *Myristicaceae* | *Virola surinamensis* | 5°17'N  52°54'W | Tropics | 2008, 3 - 7 | Unknown | 24.50 | 1.36 | (Stahl *et al.*, 2011a) |
| *Angiospermae* | *Myristicaceae* | *Iryanthera sagotiana* | 5°17'N  52°54'W | Tropics | 2008, 3 - 7 | Unknown | 24.50 | 1.13 | (Stahl *et al.*, 2011a) |
| *Angiospermae* | *Myristicaceae* | *Virola koschnyi* | 10°26'N  84°W | Tropics | 2009 - 2010 | Unknown | 25.00 | 1.00 | (Asao *et al.*, 2015) |
| *Angiospermae* | *Myristicaceae* | *Virola michelii* | 5°17'N  52°54'W | Tropics | 2008, 9 - 12 | Unknown | 25.60 | 0.89 | (Stahl *et al.*, 2011a) |
| *Angiospermae* | *Myristicaceae* | *Iryanthera sagotiana* | 5°17'N  52°54'W | Tropics | 2008, 9 - 12 | Unknown | 25.60 | 0.84 | (Stahl *et al.*, 2011a) |
| Angiospermae | Myristicaceae | *Virola cf* | 4°7'S  78°58'W | Tropics | 2005, 10 - 12 | Unknown | 20.80 | 0.43 | (Zach *et al.*, 2008) |
| Angiospermae | Rosaceae | *Bocoa prouacensis* | 5°17'N  52°54'W | Tropics | 2008, 3 - 7 | Unknown | 24.50 | 1.56 | (Stahl *et al.*, 2011a) |
| Angiospermae | Rosaceae | *Eschweilera coriacea* | 5°17'N  52°54'W | Tropics | 2008, 3 - 7 | Unknown | 24.50 | 1.26 | (Stahl *et al.*, 2011a) |
| Angiospermae | Rosaceae | *Bocoa prouacensis* | 5°17'N  52°54'W | Tropics | 2008, 9 - 12 | Unknown | 25.60 | 0.83 | (Stahl *et al.*, 2011a) |
| Angiospermae | Rosaceae | *Eschweilera coriacea* | 5°17'N  52°54'W | Tropics | 2008, 9 - 12 | Unknown | 25.60 | 0.63 | (Stahl *et al.*, 2011a) |
| Angiospermae | Styracaceae | *Styrax foveolaria* | 4°6'S  79°10'W | Tropics | 2005, 10 - 12 | Unknown | 10.60 | 0.44 | (Zach *et al.*, 2008) |
| Angiospermae | Annonaceae | *Xylopia nitida* | 5°17'N  52°54'W | Tropics | 2008, 9 - 12 | Unknown | 25.60 | 1.11 | (Stahl *et al.*, 2011a) |
| Angiospermae | Annonaceae | *Xylopia nitida* | 5°17'N  52°54'W | Tropics | 2008, 3 - 7 | Unknown | 24.50 | 0.91 | (Stahl *et al.*, 2011a) |
| Angiospermae | Vochysiaceae | *Vochysia guatemalensis* | 10°26'N  84°W | Tropics | 2009 - 2010 | Unknown | 25.00 | 0.97 | (Asao *et al.*, 2015) |
| Angiospermae | Solanaceae | *Sloanea grandiflora* | 5°17'N  52°54'W | Tropics | 2008, 3 - 7 | Unknown | 24.50 | 0.95 | (Stahl *et al.*, 2011a) |
| Angiospermae | Solanaceae | *Sloanea grandiflora* | 5°17'N  52°54'W | Tropics | 2008, 9 - 12 | Unknown | 25.60 | 0.93 | (Stahl *et al.*, 2011a) |
| Angiospermae | Dichapetalaceae | *Tapura capitulifera* | 5°17'N  52°54'W | Tropics | 2008, 3 - 7 | Unknown | 24.50 | 1.07 | (Stahl *et al.*, 2011a) |
| Angiospermae | Dichapetalaceae | *Tapura capitulifera* | 5°17'N  52°54'W | Tropics | 2008, 9 - 12 | Unknown | 25.60 | 0.75 | (Stahl *et al.*, 2011a) |
| Angiospermae | Elaeocarpaceae | *Sloanea sp.* | 5°17'N  52°54'W | Tropics | 2008, 3 - 7 | Unknown | 24.50 | 0.94 | (Stahl *et al.*, 2011a) |
| Angiospermae | Elaeocarpaceae | *Sloanea sp.* | 5°17'N  52°54'W | Tropics | 2008, 9 - 12 | Unknown | 25.60 | 0.76 | (Stahl *et al.*, 2011a) |
| Angiospermae | Meliaceae | *Carapa procera* | 5°17'N  52°54'W | Tropics | 2008, 3 - 7 | Unknown | 24.50 | 1.00 | (Stahl *et al.*, 2011a) |
| Angiospermae | Meliaceae | *Carapa procera* | 5°17'N  52°54'W | Tropics | 2008, 9 - 12 | Unknown | 25.60 | 0.67 | (Stahl *et al.*, 2011a) |
| Angiospermae | Euphorbiaceae | *Chaetocarpus schomburgkianus* | 5°17'N  52°54'W | Tropics | 2008, 3 - 7 | Unknown | 24.50 | 1.37 | (Stahl *et al.*, 2011a) |
| Angiospermae | Euphorbiaceae | *Chaetocarpus schomburgkianus* | 5°17'N  52°54'W | Tropics | 2008, 9 - 12 | Unknown | 25.60 | 0.71 | (Stahl *et al.*, 2011a) |
| Angiospermae | Euphorbiaceae | *Alchornea sp* | 4°7′S  78°58′W | Tropics | 2005, 10 - 12 | Unknown | 20.80 | 0.25 | (Zach *et al.*, 2008) |
| Angiospermae | Cunoniaceae | *Weinmannia loxensis* | 4°6'S  79°10'W | Tropics | 2005, 10 - 12 | Unknown | 10.60 | 0.32 | (Zach *et al.*, 2008) |
| Angiospermae | Symplocaceae | *Symplocos sp* | 4°6'S  79°10'W | Tropics | 2005, 10 - 12 | Unknown | 10.60 | 0.30 | (Zach *et al.*, 2008) |
| Angiospermae | Bignoniaceae | *Tabebuia insignis* | 5°17'N  52°54'W | Tropics | 2008, 3 - 7 | Unknown | 24.50 | 0.85 | (Stahl *et al.*, 2011a) |
| Angiospermae | Bignoniaceae | *Tabebuia insignis* | 5°17'N  52°54'W | Tropics | 2008, 9 - 12 | Unknown | 25.60 | 0.51 | (Stahl *et al.*, 2011a) |
| Angiospermae | Chloranthaceae | *Hedyosmum sp* | 4°6'S  79°10'W | Tropics | 2005, 10 - 12 | Unknown | 10.60 | 0.23 | (Zach *et al.*, 2008) |
| Angiospermae | Cecropiaceae | *Cecropia obtusa* | 5°17'N  52°54'W | Tropics | 2008, 3 - 7 | Unknown | 24.50 | 0.54 | (Stahl *et al.*, 2011a) |
| Angiospermae | Cecropiaceae | *Cecropia obtusa* | 5°17'N  52°54'W | Tropics | 2008, 9 - 12 | Unknown | 25.60 | 0.39 | (Stahl *et al.*, 2011a) |
| Angiospermae | Rubiaceae | *Ladenbergia cf oblongifolia* | 3°58'S  79°4'W | Tropics | 2005, 10 - 12 | Unknown | 17.20 | 0.28 | (Zach *et al.*, 2008) |
| Angiospermae | Rubiaceae | *Faramea sp* | 4°6'S  79°10'W | Tropics | 2005, 10 - 12 | Unknown | 10.60 | 0.21 | (Zach *et al.*, 2008) |
| Angiospermae | Aquifoliaceae | *Ilex weberlingii* | 4°6'S  79°10'W | Tropics | 2005, 10 - 12 | Unknown | 10.60 | 0.18 | (Zach *et al.*, 2008) |
| Angiospermae | Aquifoliaceae | *Ilex cf amboroica* | 3°58'S  79°4'W | Tropics | 2005, 10 - 12 | Unknown | 17.20 | 0.18 | (Zach *et al.*, 2008) |
| Angiospermae | Primulaceae | *Myrsine coriacea* | 3°58'S  79°4'W | Tropics | 2005, 10 - 12 | Unknown | 17.20 | 0.14 | (Zach *et al.*, 2008) |
| Angiospermae | Primulaceae | *Myrsine sp* | 4°6'S  79°10'W | Tropics | 2005, 10 - 12 | Unknown | 10.60 | 0.11 | (Zach *et al.*, 2008) |
| Angiospermae | Araliaceae | *Schefflera sp* | 3°58'S  79°4'W | Tropics | 2005, 10 - 12 | Unknown | 17.20 | 0.09 | (Zach *et al.*, 2008) |

Table S2 Stem cuticular photosynthesis of different species

| Phylum | Family | Species | Chlorophyll (mg chl m^-2^) | Photosynthetic rates | Reference |
| --- | --- | --- | --- | --- | --- |
| Angiospermae | Aceraceae | *Acer campestre* | 200 | 77 % | (Pfanz *et al.*, 2002) |
| Angiospermae | Aceraceae | *Acer platanoides* | 300 | 77 % | (Pfanz *et al.*, 2002) |
| Angiospermae | Aceraceae | *Acer pseudo-platanus* | 350 | 77 % | (Pfanz *et al.*, 2002) |
| Angiospermae | Hippocastanaceae | *Aesculus hippocastanum* | 204.5 | 77 % | (Pfanz *et al.*, 2002) |
| Angiospermae | Betulaceae | *Betula pendula* | 178.5 | 77 % | (Pfanz *et al.*, 2002) |
| Angiospermae | Fagaceae | *Fagus sylvatica* | 150 | 77 % | (Pfanz *et al.*, 2002) |
| Angiospermae | Oleaceae | *Fraxinus excelsior* | 500 | 77 % | (Pfanz *et al.*, 2002) |
| Angiospermae | Salicaceae | *Populus tremula* | 394 | 77 % | (Pfanz *et al.*, 2002) |
| Angiospermae | Fagaceae | *Quercus robur* | 417 | 77 % | (Pfanz *et al.*, 2002) |
| Angiospermae | Salicaceae | *Salix fragilis* | 300 | 77 % | (Pfanz *et al.*, 2002) |
| Angiospermae | Caprifoliaceae | *Sambucus nigra* | 300 | 77 % | (Pfanz *et al.*, 2002) |
| Angiospermae | Rosaceae | *Sorbus aucuparia* | 400 | 77 % | (Pfanz *et al.*, 2002) |
| Angiospermae | Tiliaceae | *Tilia platyphyllos* | 250 | 77 % | (Pfanz *et al.*, 2002) |
| Angiospermae | Ulmaceae | *Ulmus glabra* | 300 | 77 % | (Pfanz *et al.*, 2002) |
| Gymnospermae | Pinaceae | *Larix decidua* | 312 | 77 % | (Pfanz *et al.*, 2002) |
| Gymnospermae | Pinaceae | *Picea abies* | 225 | 77 % | (Pfanz *et al.*, 2002) |
| Gymnospermae | Pinaceae | *P. pungens* | 255.5 | 77 % | (Pfanz *et al.*, 2002) |
| Gymnospermae | Pinaceae | *Pinus nigra* | 231.5 | 77 % | (Pfanz *et al.*, 2002) |
| Gymnospermae | Pinaceae | *Larix gmelini* | 230.5 | 1.11 μmol (O_2_) m^-2^ s^-1^ | (Ren *et al.*, 2009) |
| Gymnospermae | Pinaceae | *Pinus sylvestris* | 230.5 | 0.96 μmol (O_2_) m^-2^ s^-1^ | (Ren *et al.*, 2009) |
| Gymnospermae | Pinaceae | *Pinus sylvestris* | 198 | 24.88 % | (Tarvainen *et al.*, 2018) |
| Angiospermae | Juglandaceae | *Juglans mandshurica* | 230.5 | 1.73 μmol (O_2_) m^-2^ s^-1^ | (Ren *et al.*, 2009) |
| Angiospermae | Rutaceae | *Phellodendron amurense* | 230.5 | 1.78 μmol (O_2_) m^-2^ s^-1^ | (Ren *et al.*, 2009) |
| Gymnospermae | Pinaceae | *Pnius koraiensis* | 230.5 | 0.21 μmol (O_2_) m^-2^ s^-1^ | (Ren *et al.*, 2009) |
| Gymnospermae | Pinaceae | *Pinus tabulaeformis* | 230.5 | 0.52 μmol (O_2_) m^-2^ s^-1^ | (Ren *et al.*, 2009) |
| Angiospermae | Oleaceae | *Fraxinus mandshurica* | 230.5 | 2.06 μmol (O_2_) m^-2^ s^-1^ | (Ren *et al.*, 2009) |
| Angiospermae | Betulaceae | *Betula platyphylla* | 230.5 | 1.41 μmol (O_2_) m^-2^ s^-1^ | (Ren *et al.*, 2009) |
| Angiospermae | Salicaceae | *Salix matsudana* | 230.5 | 1.57 μmol (O_2_) m^-2^ s^-1^ | (Ren *et al.*, 2009) |
| Angiospermae | Sapindaceae | *Acer saccharum* | 230.5 | 1.41 μmol (O_2_) m^-2^ s^-1^ | (Ren *et al.*, 2009) |
| Angiospermae | Myrtaceae | *Eucalyptus globulus* | 230.523 | 80.61 % | (Eyles *et al.*, 2009) |
| Angiospermae | Myricaceae | *Myrica cerifera* | 111.43 | 59.93 % | (Vick & Young, 2009) |
| Angiospermae | Salicaceae | *Populus* deltoides |  | 47.38 % | (Bloemen *et al.*, 2016) |
| Angiospermae | Betulaceae | *Alnus glutinosa* |  | 3.73 μmol m^-2^ s^-1^ | (Berveiller *et al.*, 2007) |
| Angiospermae | Oleaceae | *Fraxinus excelsior* |  | 1.06 μmol m^-2^ s^-1^ | (Berveiller *et al.*, 2007) |
| Angiospermae | Tiliaceae | *Tilia cordata* |  | 1.42 μmol m^-2^ s^-1^ | (Berveiller *et al.*, 2007) |

**A MM, Teskey RO**. **2004**. Estimating stem respiration in trees by a mass balance approach that accounts for internal and external fluxes of CO_2_. *Tree Physiology*: 5.

**Adu-Bredu S et al**. Long-term Respiratory Cost of Maintenance and Growth of Field-grown Young Hinoki Cypress (Chamaecyparis obtusa). : 6.

**Angert A, Muhr J, Negron Juarez R, Alegria Muñoz W, Kraemer G, Ramirez Santillan J, Barkan E, Mazeh S, Chambers JQ, Trumbore SE**. **2012**. Internal respiration of Amazon tree stems greatly exceeds external CO_2_ efflux. *Biogeosciences* **9**: 4979–4991.

**Araki MG, Kajimoto T, Han Q, Kawasaki T, Utsugi H, Gyokusen K, Chiba Y**. **2015**. Effect of stem radial growth on seasonal and spatial variations in stem CO_2_ efflux of Chamaecyparis obtusa. *Trees* **29**: 499–514.

**Asao S, Bedoya-Arrieta R, Ryan MG**. **2015**. Variation in foliar respiration and wood CO2 efflux rates among species and canopy layers in a wet tropical forest. **35**: 12.

**Ayumi K, Tomonori K, Hikaru K, Mizue O, Kazuho M, Ryuji I, Tomo’omi K, Kyoichi O**. **2014**. Vertical variations in wood CO_2_ efflux for live emergent trees in a Bornean tropical rainforest. *Tree Physiology*: 5.

**Berveiller D, Kierzkowski D, Damesin C**. **2007**. Interspecific variability of stem photosynthesis among tree species. *Tree Physiology* **27**: 53–61.

**Bloemen J, Vergeynst LL, Overlaet-Michiels L, Steppe K**. **2016**. How important is woody tissue photosynthesis in poplar during drought stress? *Trees* **30**: 63–72.

**Bolstad PV, Davis KJ, Martin J, Cook BD, Wang W**. **2004**. Component and whole-system respiration fluxes in northern deciduous forests. **24**: 12.

**Brito P, Morales D, Wieser G, Jimenez MS**. **2010**. Spatial and seasonal variations in stem CO2 efﬂux of Pinus canariensis at their upper distribution limit. : 9.

**Bronson DR, Gower ST**. **2010**. Ecosystem warming does not affect photosynthesis or aboveground autotrophic respiration for boreal black spruce. **30**: 9.

**Cerasoli S, McGuire MA, Faria J, Mourato M, Schmidt M, Pereira JS, Chaves MM, Teskey RO**. **2009**. CO2 efflux, CO2 concentration and photosynthetic refixation in stems of Eucalyptus globulus (Labill.). *Journal of Experimental Botany* **60**: 99–105.

**Chen X, Hutley LB, Eamus D**. Carbon balance of a tropical savanna of northern Australia. : 12.

**Chi Y, Yang Q, Zhou L, Shen R, Zheng S, Zhang Z, Zhang Z, Xu M, Wu C, Lin X, *et al.*** **2020a**. Temperature Sensitivity in Individual Components of Ecosystem Respiration Increases along the vertical gradient of Leaf–stem–soil in Three Subtropical Forests. : 15.

**Chi Y, Yang Q, Zhou L, Shen R, Zheng S, Zhang Z, Zhang Z, Xu M, Wu C, Lin X, *et al.*** **2020b**. Temperature Sensitivity in Individual Components of Ecosystem Respiration Increases along the Vertical Gradient of Leaf–Stem–Soil in Three Subtropical Forests. *Forests* **11**: 140.

**Doughty CE, Metcalfe DB, da Costa MC, de Oliveira AAR, Neto GFC, Silva JA, Aragão LEOC, Almeida SS, Quesada CA, Girardin CAJ, *et al.*** **2014**. The production, allocation and cycling of carbon in a forest on fertile *terra preta* soil in eastern Amazonia compared with a forest on adjacent infertile soil. *Plant Ecology & Diversity* **7**: 41–53.

**Edwards NT, Hanson PJ**. **1996**. Stem respiration in a closed-canopy upland oak forest. *Tree Physiology* **16**: 433–439.

**Eyles A, Pinkard EA, O’Grady AP, Worledge D, Warren CR**. **2009**. Role of corticular photosynthesis following defoliation in *Eucalyptus globulus*. *Plant, Cell & Environment* **32**: 1004–1014.

**Gaumont-Guay D, Black TA, Griffis TJ, Barr AG, Morgenstern K, Jassal RS, Nesic Z**. **2006**. Influence of temperature and drought on seasonal and interannual variations of soil, bole and ecosystem respiration in a boreal aspen stand. *Agricultural and Forest Meteorology* **140**: 203–219.

**Gielen B, Scarascia-Mugnozza G, Ceulemans R**. **2003**. Stem respiration of Populus species in the third year of free-air CO_2_ enrichment. *Physiologia Plantarum* **117**: 500–507.

**Gruber A, Wieser G, Oberhuber W**. **2009**. Intra-annual dynamics of stem CO_2_ efflux in relation to cambial activity and xylem development in Pinus cembra. *Tree Physiology* **29**: 641–649.

**Hu WY, Sha LQ**. **2010**. A study on the Stem respiration of three dominant tree species in a montane evergreen broad-leaved forest in Ailao Mountains,China. *Journal of Yunnan University* **32**: 613–620.

**Jie T, Fan H, Wang Y, Li Z**. **2017**. Stem CO_2_ Efflux Ratio of Pinus massoniana of Various DBH Classes and Its Sensitivity to Temperature. *Scientia Silvae Sinicae*.

**Khomik M, Arain MA, Brodeur JJ, Peichl M, Restrepo-Coupé N, McLaren JD**. **2010**. Relative contributions of soil, foliar, and woody tissue respiration to total ecosystem respiration in four pine forests of different ages. *Journal of Geophysical Research* **115**: G03024.

**Kim MH, Nakane K**. **2005**. Effects of flow rate and chamber position on measurement of stem respiration rate with an open flow system in a Japanese red pine. *Forest Ecology and Management* **210**: 469–476.

**Law BE, Ryan MG, Anthoni PM**. **1999**. Seasonal and annual respiration of a ponderosa pine ecosystem. *Global Change Biology* **5**: 169–182.

**Marler TE**. **2020**. Stem CO_2_ efflux of *Cycas micronesica* is reduced by chronic non-native insect herbivory. *Plant Signaling & Behavior* **15**: 1716160.

**Miao W, Lanzhu JI, Qiurong LI, Dongmei X, Hailiang LIU**. **2005**. Stem respiration of Pinus koraiensis in Changbai Mountains. *Chinese Journal of Applied Ecology* **16**: 7–13.

**Miyama T, Kominami Y, Tamai K, Goto Y, Kawahara T, Jomura M, Dannoura M**. **2006**. Components and seasonal variation of night-time total ecosystem respiration in a Japanese broad-leaved secondary forest. *Tellus B: Chemical and Physical Meteorology* **58**: 550–559.

**Pfanz H, Aschan G, Langenfeld-Heyser R, Wittmann C, Loose M**. **2002**. Ecology and ecophysiology of tree stems: corticular and wood photosynthesis. *Naturwissenschaften* **89**: 147–162.

**Ren F, Sun G, Hu Y, Al E**. **2009**. A preliminary studies on photosynthetic characteristics of Chlorenchyma in several tree barks. *Plant Physiology Communications* **45**: 249－252.

**Rodríguez-Calcerrada J, Martin-StPaul NK, Lempereur M, Ourcival J-M, Rey M del C del, Joffre R, Rambal S**. **2014**. Stem CO_2_ efflux and its contribution to ecosystem CO2 efflux decrease with drought in a Mediterranean forest stand. *Agricultural and Forest Meteorology* **195–196**: 61–72.

**Ryan MG, Gower ST, Hubbard RM, Waring RH, Gholz HL, Cropper WP, Running SW**. **1995a**. Woody tissue maintenance respiration of four conifers in contrasting climates. *Oecologia* **101**: 133–140.

**Ryan MG, Gower ST, Hubbard RM, Waring RH, Gholz HL, Cropper WP, Running SW**. **1995b**. Woody tissue maintenance respiration of four conifers in contrasting climates. *Oecologia* **101**: 133–140.

**Salomón RL, De Roo L, Oleksyn J, De Pauw DJW, Steppe K**. **2020**. TR eSpire – a biophysical TR ee Stem respiration model. *New Phytologist* **225**: 2214–2230.

**Salomón RL, Valbuena-Carabaña M, Gil L, McGuire MA, Teskey RO, Aubrey DP, González-Doncel I, Rodríguez-Calcerrada J**. **2016**. Temporal and spatial patterns of internal and external stem CO_2_ fluxes in a sub-Mediterranean oak (M Ryan, Ed.). *Tree Physiology*: tpw029.

**Stahl C, Burban B, Goret J-Y, Bonal D**. **2011a**. Seasonal variations in stem CO_2_ efflux in the Neotropical rainforest of French Guiana. *Annals of Forest Science* **68**: 771–782.

**Stahl C, Burban B, Goret J-Y, Bonal D**. **2011b**. Seasonal variations in stem CO_2_ efflux in the Neotropical rainforest of French Guiana. *Annals of Forest Science* **68**: 771–782.

**Stahl C, Burban B, Goret J-Y, Bonal D**. **2011c**. Seasonal variations in stem CO_2_ efflux in the Neotropical rainforest of French Guiana. *Annals of Forest Science* **68**: 771–782.

**Tarvainen L, Wallin G, Lim H, Linder S, Oren R, Ottosson Löfvenius M, Räntfors M, Tor-ngern P, Marshall J**. **2018**. Photosynthetic refixation varies along the stem and reduces CO2 efflux in mature boreal Pinus sylvestris trees. *Tree Physiology* **38**: 558–569.

**Ubierna N, Kumar AS, Cernusak LA, Pangle RE, Gag PJ, Marshall JD**. **2009**. Storage and transpiration have negligible eﬀects on d^13^C of stem CO_2_ eﬄux in large conifer trees. **29**: 12.

**Vick JK, Young DR**. **2009**. Corticular photosynthesis: A mechanism to enhance shrub expansion in coastal environments. *Photosynthetica* **47**: 26–32.

**Wang M, Ji LZ, Li QR, Xiao DM, Liu HL**. **2005**. Stem respiration of Pinus koraiensis in Changbai Mountains. *Chinese Journal of Applied Ecology*: 7–13.

**Wang XW, Mao ZJ, Forestry SO, University NF**. **2014**. CO_2_ Flux Components,Their Contributions to Stem Respiration and the Key Impact Factors in Stems of Larch. *Bulletin of Botanical Research* **34**: 452–457.

**Wang X, Mao Z, McGuire MA, Teskey RO**. **2019**. Stem radial CO_2_ conductance affects stem respiratory CO2 fluxes in ash and birch trees. *Journal of Forestry Research* **30**: 21–29.

**Wang X, Sun T, Wu J**. **2011**. Effects of temperature and sap flow velocity on CO2 efflux from stems of three tree species in spring and autumn in Northeast China. *Chinese Journal of Ecology* **31**: 3358–3367.

**Wang W, Yang F, Zu Y, Wang H, TAKAGI K, SASA K, Takayoshi K**. **2003**. Stem Respiration of a Larch (*Larix gmelini*) Plantation in Northeast China. *Acta Botanica Sinica* **45**: 1387–1396.

**Wei GJ, Sheng H, Yang ZJ, Yuan YD, Yang YS**. **2009**. Diurnal Dynamics of CO_2_ Released from Bole Surface of Four Street Tree Species in Subtropical Region of China. *Journal of Subtropical Resources and Environment*: 23–31.

**Wieser G, Bahn M**. **2004**. Seasonal and spatial variation of woody tissue respiration in a Pinus cembra tree at the alpine timberline in the central Austrian Alps. *Trees* **18**.

**Xiao F, Xiong C, Zhang X, Qi L, Zhang T, Xu W, Xu H**. **2010**. A measurement of moso bamboo plantation community respiration. In: International Conference on the Efforts in Response to Forest-related Natural Disasters-forest Science Frum.

**Xu F, Wang CK, Wang XC**. **2011**. Intra- and inter-specific variations in stem respiration for 14 temperate tree species in northeastern China. *Acta Ecologica Sinica*: 3581–3589.

**Yang Q, Liu L, Zhang W, Xu M, Wang S**. **2015**. Different responses of stem and soil CO2 efflux to pruning in a Chinese fir (Cunninghamia lanceolata) plantation. *Trees* **29**: 1207–1218.

**Yang Y, Zhao M, Xu X, Sun Z, Yin G, Piao S**. **2014**. Diurnal and Seasonal Change in Stem Respiration of Larix principis-rupprechtii Trees, Northern China (D Hui, Ed.). *PLoS ONE* **9**: e89294.

**Zach A, Horna V, Leuschner C**. **2008**. Elevational change in woody tissue CO2 efflux in a tropical mountain rain forest in southern Ecuador. *Tree Physiology* **28**: 67–74.

**Zachariah EJ, Sabulal B, Nair DNK, Johnson AJ, Kumar CSP**. **2016**. Carbon dioxide emission from bamboo culms. *Plant Biology*: 6.

**Zha T**. **2004**. Seasonal and Annual Stem Respiration of Scots Pine Trees under Boreal Conditions. *Annals of Botany* **94**: 889–896.

**Zhao G, Liu G, Zhu W**. **2018**. Spatial variations in the stem CO_2_ efflux rate of *Abies fabri* and the response to temperature in the Gongga Mountains. *Acta Ecologica Sinica* **38**.


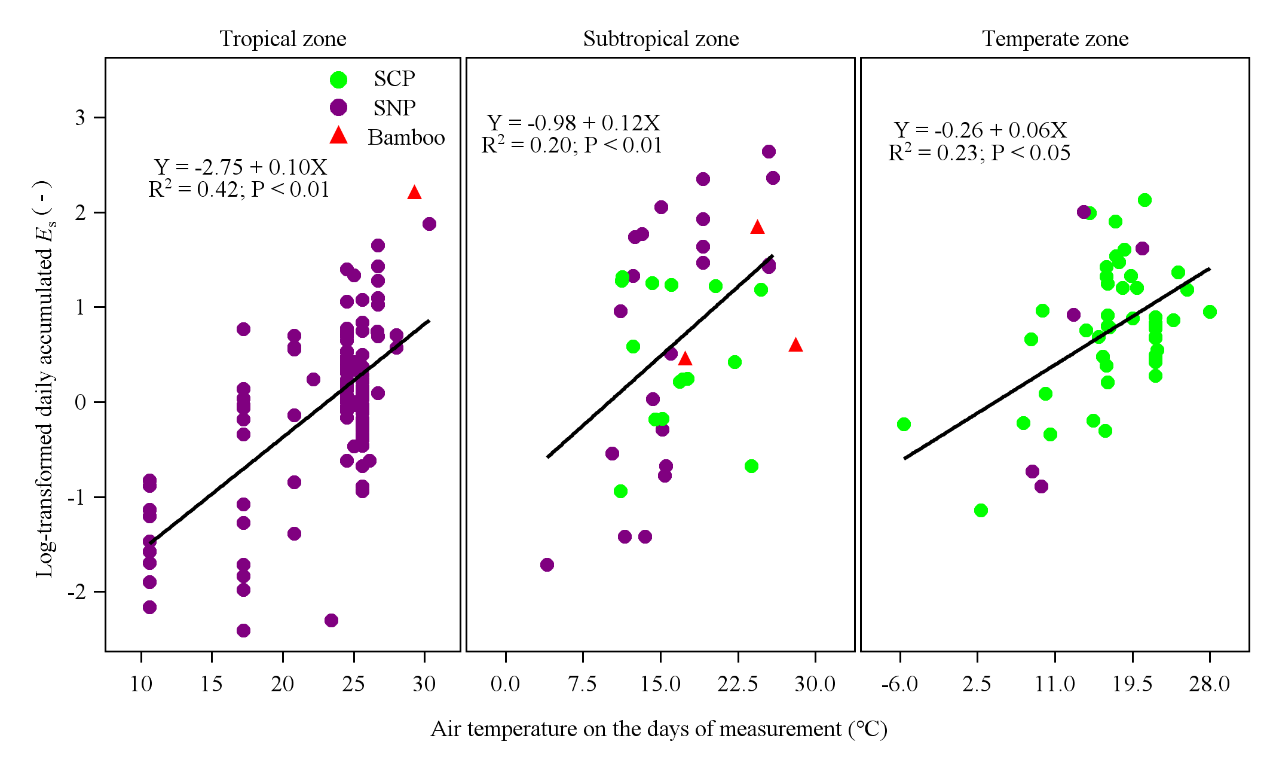


**Figure S1.** Relationship between temperature and stem surface CO_2_ efflux across species with cuticular photosynthesis (SCP), without cuticular photosynthesis (SNP), and bamboos in different climate zones (191 species included) on the days of measurement.
